# Curcumin-Based Tyrosinase-Targeting Derivatives for Cell Imaging and Depigmenting Effect

**DOI:** 10.3390/ijms27198759

**Published:** 2026-09-30

**Authors:** Maura Monforte, Marilena Pontoriero, Paola Maria Bonaccorsi, Antonella Calderaro, Leonardo Centorrino, Valeria D’Angelo, Laura De Luca, Maria Paola Germanò, Rosaria Gitto, Salvatore Mirabile, Giuseppe Tancredi Patanè, Stefano Putaggio, Domenica Scumaci, Chiara Maria Antonietta Gangemi

**Affiliations:** 1 Department of Chemical, Biological, Pharmaceutical, and Environmental Sciences, University of Messina, Viale F. Stagno d’Alcontres 31, 98166 Messina, Italylaura.deluca@unime.it (L.D.L.);; 2 Proteomics and Tumor Biochemistry Group, Department of Experimental and Clinical Medicine, School of Medicine, Magna Græcia University of Catanzaro, Viale Europa, 88100 Catanzaro, Italyscumaci@unicz.it (D.S.); 3 Fondazione “Prof. Antonio Imbesi”, 98123 Messina, Italy

**Keywords:** curcumin derivatives, tyrosinase, depigmenting, fluorescence imaging, antioxidant

## Abstract

Targeting melanogenesis through tyrosinase inhibition represents a key therapeutic strategy, highlighting the need for novel molecules capable of modulating melanin synthesis and/or serving as fluorescent probes for cellular and melanoma tracking. In this study, two curcumin (CUR) derivatives, **CUR-C3-PP 1** and **CUR-CO-PP 2**, were designed and successfully synthesized by strategic integration of a 4-(piperazin-1-yl)phenol pharmacophore into CUR skeleton. In vitro enzymatic assays against *Agaricus bisporus* tyrosinase (*Ab*TYR) revealed that derivatives **CUR-C3-PP 1** and **CUR-CO-PP 2** amplified the diphenolase inhibitory capacity (IC_50_ values of 51.20 μM and 12.89 μM, respectively) when compared to parent curcumin (IC_50_ = 168.57 μM). Notably, **CUR-CO-PP 2** emerged as the most potent candidate, significantly eclipsing native CUR and outperforming the standard inhibitor kojic acid (KA). Enzyme kinetics and molecular docking simulations rationalized this enhanced profile, and the mechanism of inhibitory effects for compounds **CUR-C3-PP 1** and **CUR-CO-PP 2**. Cellular assays highlighted that **CUR-C3-PP 1** retained functional intrinsic luminescence, displaying selective accumulation and intense fluorescence within the cytoplasm of B16-F10 melanoma cells alongside preferential cytotoxicity. Screening in zebrafish (*Danio rerio*) embryos confirmed that both derivatives maintain an excellent biocompatibility profile up to 20 μM. In vivo, both compounds elicited robust, dose-dependent depigmentation, with **CUR-CO-PP 2** closely approaching the efficacy of the control 1-phenyl-2-thiourea (PTU). Collectively, our findings demonstrate that the imaging and anti-melanogenic properties of curcuminoids can be independently optimized through rational linker modification, establishing **CUR-C3-PP 1** as a promising fluorescent tracer for imaging and **CUR-CO-PP 2** as a candidate for depigmenting therapies.

## 1. Introduction

Skin pigmentation is primarily determined by melanin synthesis and is modulated by genetic background, ultraviolet (UV) exposure, aging, hormonal status, and certain medications. Pigmentary disorders are broadly classified as hyperpigmentation (Melasma, Acanthosis nigricans, Hemochromatosis, Post-inflammatory hyperpigmentation) or hypopigmentation (Vitiligo, Pityriasis alba, Chemical leukoderma). Hyperpigmentation is a localized or diffuse skin darkening driven by elevated melanin deposition, vascular dynamics, epidermal thickness, and heavy metal exposure [1], and profoundly impacts patient quality of life [2]. Melasma is a prevalent chronic form, presenting UV-induced facial macules with a higher incidence in women due to pregnancy and oral contraceptives. Its pathogenesis involves melanocyte hyperactivity mediated by microphthalmia-associated transcription factor (MITF) and tyrosinase (TYR) upregulation, light-induced signaling, and estrogen/progesterone pathways [3]. Notably, hyperpigmentation and melanoma share melanocytic origins, involving overlapping cellular pathways that dictate both benign and malignant pigmentary phenotypes [4].

Melanogenesis is an enzymatically regulated process through which melanocytes synthesize melanin, which is subsequently stored in melanosomes and determines skin, hair, and eye pigmentation. This process can be modulated through multiple mechanisms, including direct inhibition of tyrosinase catalytic activity and suppression of key signaling pathways such as the α-MSH–MC1R axis, Wnt, PI3K/Akt, and MAPK cascades. These pathways converge on MITF, leading to reduced expression of its downstream targets TYR, Tyrosinase-Related Protein 1 (TRP-1), and Tyrosinase-Related Protein 2 (TRP-2). Additional regulatory strategies include antioxidant-mediated attenuation of oxidative stress, inhibition of melanocyte proliferation, and disruption of melanosome biogenesis and intracellular transport, collectively reducing melanin synthesis and accumulation [5]. These mechanistic insights establish a robust framework for the rational development of chemical and pharmacological modulators of skin pigmentation.

A wide range of phytochemicals and plant extracts exert anti-melanogenic effects through three principal mechanisms: direct inhibition of TYR catalytic activity, transcriptional downregulation of MITF, and UV-induced signaling cascades. Despite promising results in cellular and animal models, only a limited collection of natural products has shown clinically validated depigmenting activity in human clinical trials [6]. Among them, turmeric (*Curcuma longa*) extract has attracted considerable attention for its capacity to regulate melanogenesis. Curcumin (CUR), its principal bioactive polyphenol, exhibits potent anti-inflammatory and antioxidant properties that mitigate oxidative stress–driven pigmentation disorders, including melasma [7]. It has been suggested that CUR suppresses melanogenesis by inhibiting TYR expression and activity [7], by modulating intracellular signaling cascades, including activation of the Akt pathway, which leads to reduced MITF transcriptional activity in both murine B16 melanoma cells and human melanocytes [8,9], Owing to its favorable safety profile and pleiotropic mechanism of action, CUR has also been identified as a promising polyphenolic scaffold in melanoma chemoprevention and adjunct therapy [10]. Noteworthy, structurally modified CUR derivatives and curcuminoids have demonstrated enhanced anti-hyperpigmentation efficacy, often displaying improved chemical stability and stronger TYR inhibitory activity compared with the parent compound, highlighting structure–activity relationships that support their development as next-generation depigmenting agents [11].

**Chart 1:**
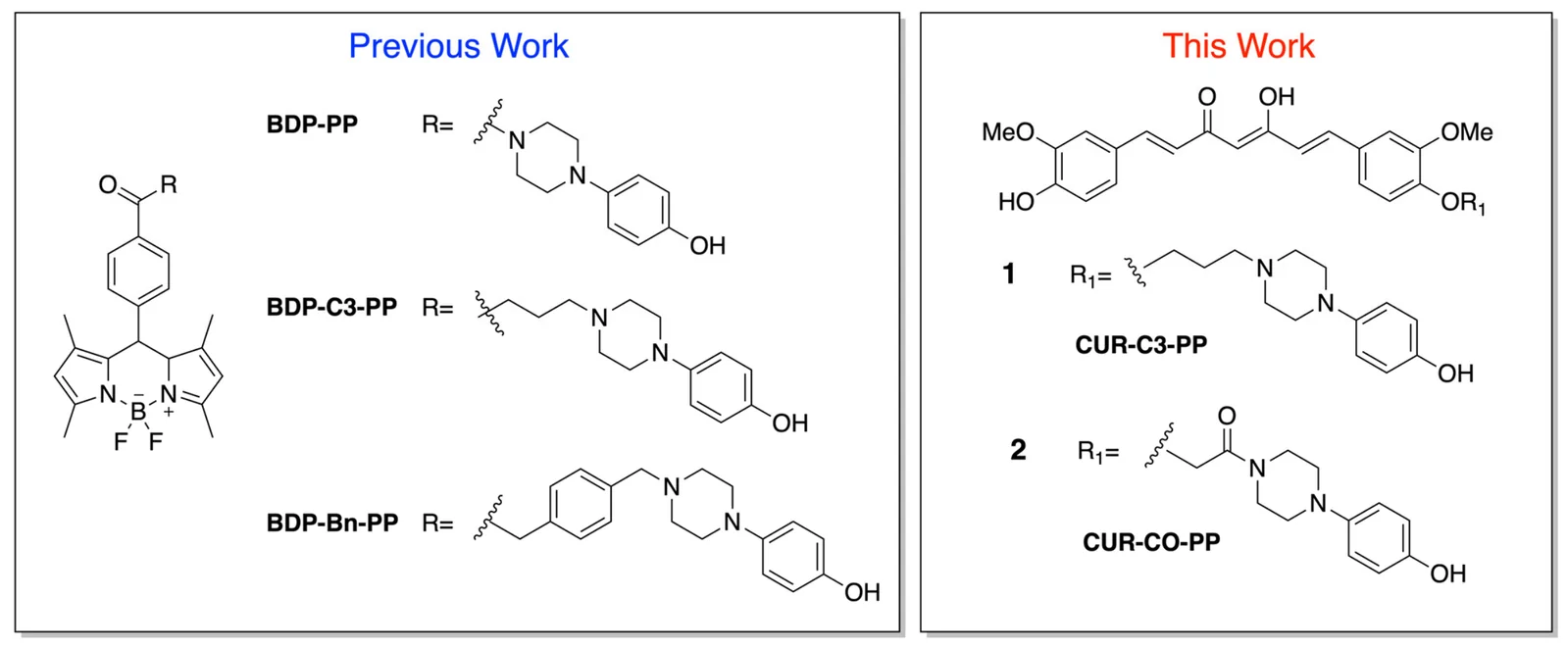
Chemical structures of luminescent 4-(piperazin-1-yl)phenol-based derivatives.

In a previous study [12], we have reported the design and synthesis of a novel class of BODIPY-based 4-(piperazin-1-yl)phenol (**PP**) derivatives (Chart 1, left side), in which the well-established TYR inhibitory activity of the piperazinyl phenolic scaffold [13] was strategically integrated with the intrinsic luminescent properties of the BODIPY core [14]. This hybrid molecular architecture was conceived to generate fluorescently traceable inhibitors capable of exerting anti-melanogenic effects while simultaneously enabling the visualization of their biodistribution and biocompatibility in biological systems. In vitro investigations have demonstrated that the BODIPY-conjugated derivatives were non-cytotoxic and exhibited marked inhibitory activity against the monophenolase function of TYR. Notably, conjugation of the piperazinyl phenolic fragment to the BODIPY scaffold conferred luminescence and improved biocompatibility. Specifically, **BDP-C3-PP** (Chart 1, left side) has shown inhibitory effects towards TYR from *Agaricus bisporus* (*Ab*TYR) at micromolar concentration (IC_50_ values = 17.10 ± 0.84 and 70.40 ± 0.94 for mono- and diphenolase, respectively); notably, this BODIPY-based 4-(piperazin-1-yl)phenol derivative has demonstrated a pronounced depigmenting effect in zebrafish embryos, with preferential localization in the lipid-rich yolk sac, an anatomical region enriched in migratory melanocytes, responsible for melanin production during early embryogenesis.

To enlarge our investigation on TYR inhibitors bearing 4-(piperazin-1-yl)phenol moiety, in the present study, we chose to connect this key structural fragment to the CUR skeleton and designed the two piperazinyl-curcumin derivatives **CUR-C3-PP 1** and **CUR-CO-PP 2** displayed in Chart 1 (right side). We selected CUR based on its well-documented ability to modulate melanogenesis [15] as well as for its intrinsic fluorescence. Due to the highly symmetric structure of π-delocalized electrons, with an α,β-unsaturated-β-diketone moiety, CUR exhibits strong absorption in the UV/Vis region, with a maximum in the range from 400 to 440 nm, while the emission is very susceptible to the solvent (460–560 nm) with a Stokes shift between 2000 and 6000 cm^−1^ [16]. CUR exerts both a persistent luminescence and a large spectrum of therapeutic effects that are influenced by the keto-enol tautomerism of the 1,6-heptadiene-3,5-dionyl chain and has been extensively used in several bioimaging and sensing applications [17]. Therefore, we hypothesized that merging a TYR-inhibiting pharmacophore with the curcumin backbone could generate hybrid derivatives capable of exerting a synergistic effect on the regulation of melanogenesis while retaining intrinsic luminescent properties for cellular tracking. The newly synthesized CUR-based 4-(piperazin-1-yl)phenol derivatives were assayed as TYR inhibitors using *Ab*TYR; their binding modes were studied in depth through in silico investigations. To explore the biomedical potential of these systems, the in vitro investigation was extended to selected melanoma cell lines, enabling assessment of both their anti-melanogenic efficacy and their suitability as luminescent probes in a tumor-relevant context. Finally, we carried out the in vivo screening of anti-melanogenic effects as well as toxic profile by employing the zebrafish (*Danio rerio*) embryos model.

## 2. Results and Discussion

### 2.1. Chemistry

**Scheme 1:**
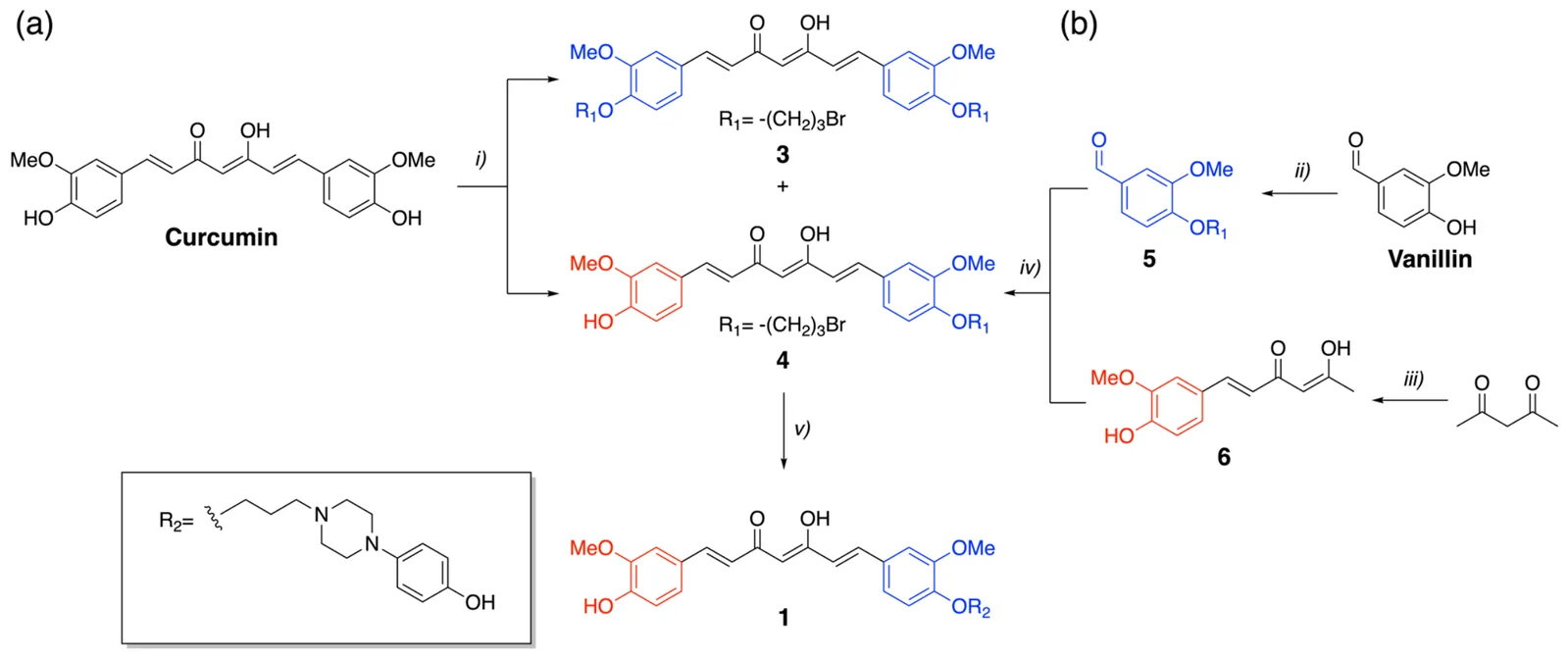
Reagents and conditions: (**a**) (*i*) Br(CH_2_)_3_Br, K_2_CO_3_, dry acetone, rt; (**b**) (*ii*) Br(CH_2_)_3_Br, K_2_CO_3_, dry DMF, 80 °C; (*iii*) B_2_O_3_, ethyl acetate, 80 °C, 30 min; then vanillin and (*n*-BuO)_3_B in ethyl acetate, 30 min, then *n*-BuNH_2_, 30 min; then 1 N HCl, 50 °C, 30 min. (*iv*) B_2_O_3_, ethyl acetate, 80 °C, 30 min, then compound **5** and (*n*-BuO)_3_B in ethyl acetate, 30 min, then piperidine, 30 min, then 0.4 N HCl, 50 °C, 30 min; (*v*) 4-(piperazin-1-yl)phenol (**PP**), NaHCO_3_, dry acetonitrile, reflux.

Two synthetic routes were pursued to obtain the designed CUR derivatives, **CUR-C3-PP 1** and **CUR-CO-PP 2**, shown in Chart 1. The synthesis of compound **CUR-C3-PP 1** was undertaken first, utilizing a linker identical to the previously developed active compound **BDP-C3-PP** (Chart 1) [12]. Curcumin was reacted with 1,3-dibromopropane in dry acetone, in the presence of K_2_CO_3_ to afford the mono-bromopropyl curcumin derivative **4** along with the symmetrically substituted compound **3** [18] in a 1:0.5 ratio. Following purification via column chromatography to separate the two curcumin derivatives, compound **4** was treated with **PP** under basic conditions (K_2_CO_3_) to yield the target compound **CUR-C3-PP 1** (Scheme 1a). To overcome the purification difficulties of compound **4** obtained in a very low yield, the synthetic pathway was optimized using an adapted Pabon methodology [19]. According to Scheme 1b, the target unsymmetrically substituted curcumin derivative **CUR-C3-PP 1** was synthesized starting from vanillin. The *O*-alkylation of vanillin with 1,3-dibromopropane in the presence of K_2_CO_3_ afforded bromopropyl aldehyde **5** in good yield [20]. Concurrently, the aldol condensation of an acetylacetone (2,4-pentanedione)–boron complex with vanillin yielded hemicurcumin **6**. Aldehyde **5** and hemicurcumin **6** were then coupled to obtain compound **4**. Although the two synthetic approaches provided comparable overall yields, the latter was preferred because it allowed simpler and more accessible chromatographic purifications. Finally, nucleophilic substitution of **PP** onto the bromine atom of compound **4** afforded **CUR-C3-PP 1** in 58% yield, after purification.

**Scheme 2:**
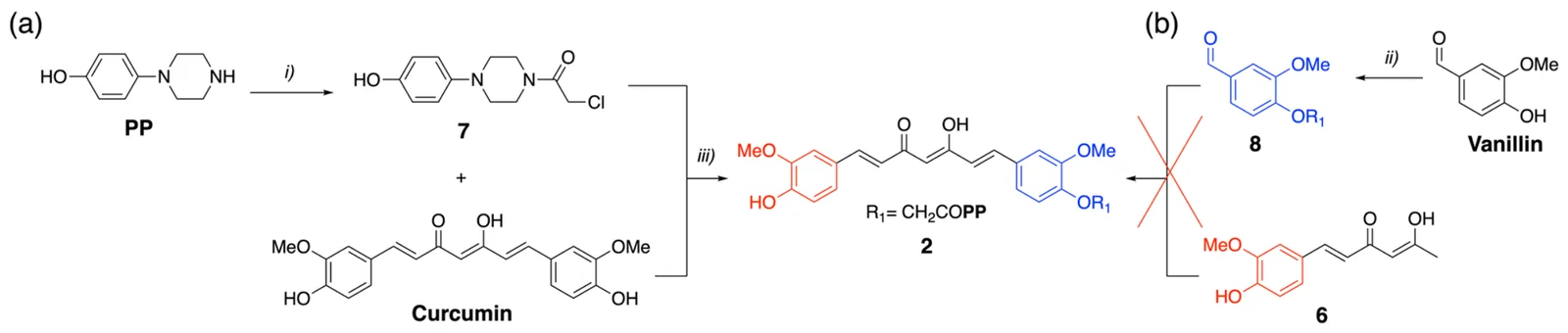
Reagents and conditions:  (**a**) (*i*) 2-Chloroacetyl chloride, dry DMF, 0 °C to rt; (*iii*) K_2_CO_3_, dry DMF, 80 °C.; (**b**) (*ii*) compound **7**, K_2_CO_3_, dry DMF, 80 °C.

For the synthesis of **CUR-CO-PP 2** (Scheme 2), starting material **PP** was reacted with 2-chloroacetyl chloride to obtain derivative **7** in good yield [21]. Vanillin was *O*-alkylated with compound **7** (Scheme 2b) and the obtained aldehyde **8** was subjected to condensation with hemicurcumin **6** in the same conditions previously applied for compound **4** but without any significant result. The strategy was then modified to investigate the direct functionalization of one of the two phenolic OH groups of curcumin with **7**, under basic conditions (Scheme 2a). This reaction successfully afforded **CUR-CO-PP 2** in 20% yield, after purification by column chromatography.

The photophysical properties of **CUR-C3-PP 1** and **CUR-CO-PP 2** were investigated in DCM and phosphate-buffered saline (PBS) containing 1% DMSO, comparing data with CUR. The corresponding absorption and emission spectra are reported in the Supplementary Materials (Figures S15–S17). Under non-aggregating conditions, monosubstitution of the CUR scaffold with the **PP** moiety resulted in only moderate changes in the photophysical properties of the derivatives.

### 2.2. Biological Evaluations

#### 2.2.1. Tyrosinase Inhibition Assay

CUR-C3-PP 1 and CUR-CO-PP 2 were initially evaluated against the monophenolase and diphenolase activities of *Ab*TYR, an established benchtop model for discovering novel anti-melanogenic agents. Initial screening of TYR inhibitors is commonly performed using AbTYR as a well-established and cost-effective model for the human enzyme (hTYR) [22]. Although, hTYR is difficult to purify and crystallize because of its transmembrane nature, AbTYR shares a similar catalytic core, making it a suitable model for preliminary screening. The enzymatic activity was monitored spectrophotometrically by measuring the rate of dopachrome formation. Specifically, L-tyrosine and L-DOPA were employed as substrates to discriminate between the mono- and diphenolase activities, respectively.

The tyrosinase inhibitory effects of the newly synthesized derivatives **CUR-C3-PP 1** and **CUR-CO-PP 2** are summarized in Table 1, alongside CUR and KA as reference standards. Regarding the monophenolase activity, compound **CUR-CO-PP 2** emerged as the most potent inhibitor among the tested molecules (IC_50_ = 19.92 μM), outperforming both CUR and KA. Conversely, compound **CUR-C3-PP 1** displayed moderate inhibition (IC_50_ = 55.34 μM); although less effective than **CUR-CO-PP 2** and KA, it marked a substantial improvement over its parent scaffold, CUR. A consistent trend was observed for the diphenolase cycle. Derivative **CUR-CO-PP 2** proved to be the most effective inhibitor (IC_50_ = 12.89 μM), surpassing the reference standard KA. Compound **CUR-C3-PP 1** also exhibited notable inhibitory effects (IC_50_ = 51.20 μM), though its potency fell short of both **CUR-CO-PP 2** and KA.

The enhanced inhibitory profile of compound **CUR-CO-PP 2** relative to **CUR-C3-PP 1** can be attributed to the structural divergence in the linker connecting the pharmacophoric moiety to the curcumin core. While **CUR-C3-PP 1** features a flexible three-carbon aliphatic chain, **CUR-CO-PP 2** incorporates an amide-bearing spacer (Chart 1). These findings suggest that the nature of the linker plays an important role in modulating the inhibitory activity of the curcumin derivatives.

#### 2.2.2. Kinetic Mechanism Analysis

**Figure 1:**
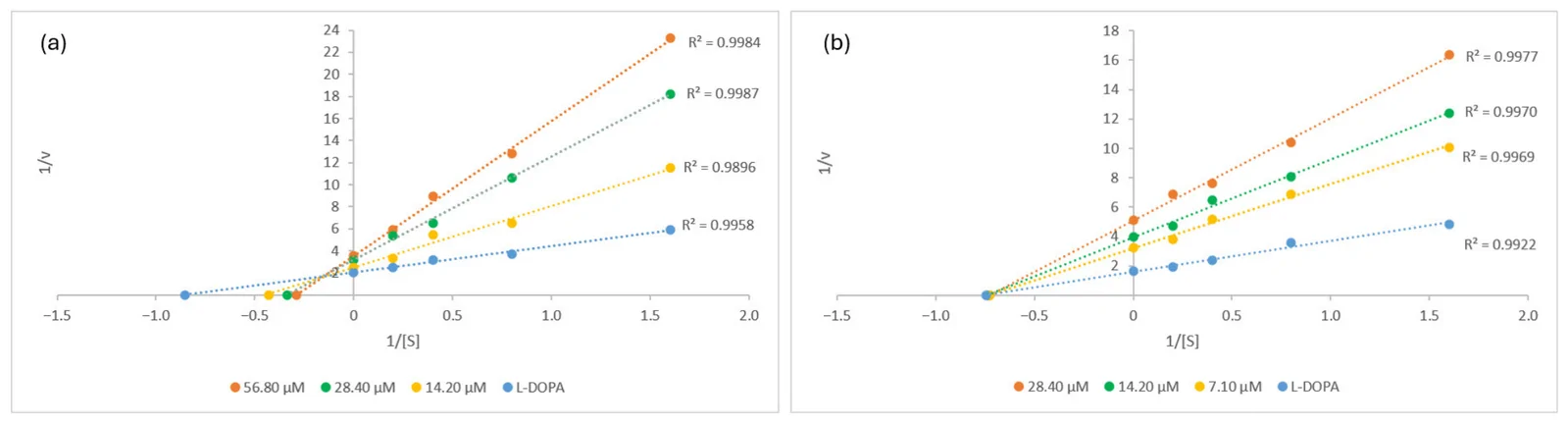
Lineweaver−Burk plots for the *Ab*TYR diphenolase inhibition using (panel (**a**)) **CUR-C3-PP 1** (14.20–56.80 µM) and (panel (**b**)) **CUR-CO-PP 2** (7.10–28.40 µM) in the presence of substrate L-Dopa (0.625–5 mM).

To elucidate the underlying mechanism of action, the kinetic behavior of *Ab*TYR diphenolase activity was investigated in the presence of derivatives **CUR-C3-PP 1** and **CUR-CO-PP 2**. Enzyme kinetics were evaluated by monitoring the reaction rate as a function of L-DOPA concentration across a range of inhibitor concentrations. Double-reciprocal Lineweaver–Burk plots (1/v vs. (1/[S]) were constructed to determine the inhibition modality (Figure 1). The resulting linear plots revealed distinct inhibition patterns for each compound. For derivative **CUR-C3-PP 1,** the plot diagnosed a mixed-type inhibition mechanism, while for compound **CUR-CO-PP 2,** a non-competitive inhibition was revealed.

#### 2.2.3. Antioxidant Assay

**Figure 2:**
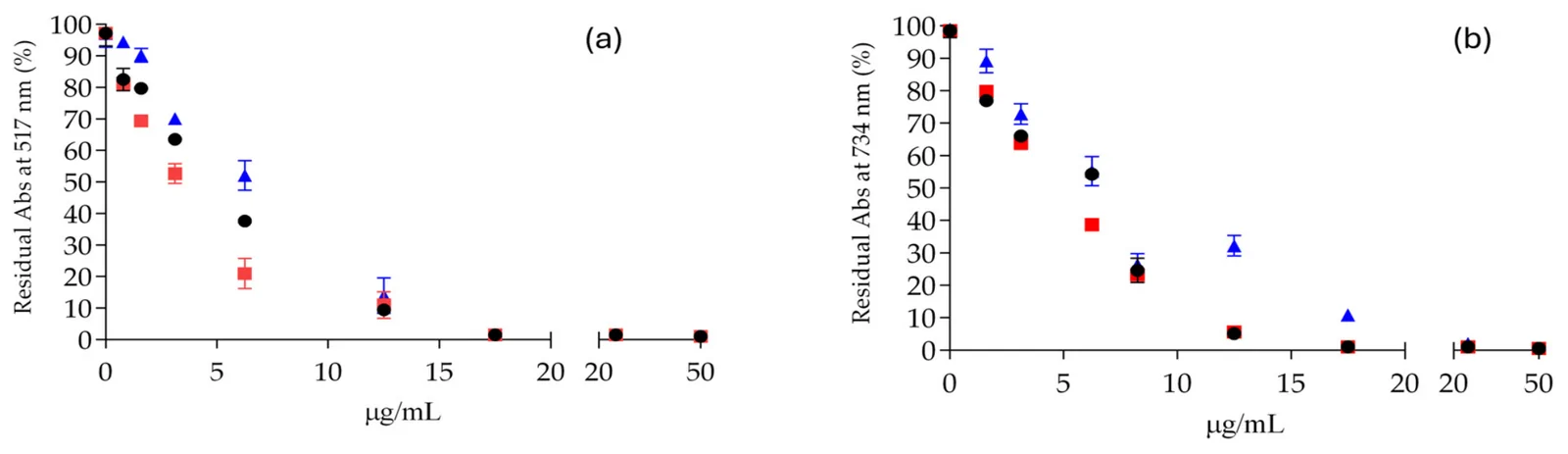
Determination of the antioxidant potentials of compounds **CUR-C3-PP 1**, **CUR-CO-PP 2,** and CUR against DPPH^●^ (**a**) and ABTS^●+^ (**b**) radicals. (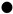) CUR; (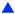) compound **CUR-C3-PP 1**; (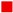) compound **CUR-CO-PP 2**.

The antioxidant properties of compounds **CUR-C3-PP 1**, **CUR-CO-PP 2,** and CUR were evaluated using the DPPH, ABTS, and FRAP assays. DPPH and ABTS assays evaluate the radical-scavenging capacity of antioxidants through the quenching of stable radical species (Figure 2), whereas the FRAP assay measures the ferric ion-reducing ability of a compound and therefore reflects its electron-donating power. The combined use of these assays provided complementary information on different mechanisms underlying antioxidant activity. In both radical-scavenging assays, lower IC_50_ values indicated stronger antioxidant activity. Compound **CUR-CO-PP 2** exhibited the highest activity, with IC_50_ values of 3.16 ± 0.34 μg mL^−1^ and 4.32 ± 0.45 μg mL^−1^ in the DPPH and ABTS assays, respectively. Compounds **CUR-C3-PP 1** and CUR showed lower antioxidant efficacy, displaying DPPH IC_50_ values of 5.67 ± 0.78 and 4.56 ± 0.62 μg mL^−1^, respectively, and ABTS IC_50_ values of 6.15 ± 0.51 and 5.04 ± 0.72 μg mL^−1^, respectively. Overall, the activity trend observed in both assays was **CUR-CO-PP 2** > CUR > **CUR-C3-PP 1**, indicating a consistent superiority of compound **CUR-CO-PP 2** in quenching free radicals. Considering the obtained values, the difference between compounds **CUR-CO-PP 2** and **CUR-C3-PP 1**, as well as between compounds **CUR-CO-PP 2** and CUR, appears evident, particularly in the DPPH assay, whereas the activities of compounds **CUR-C3-PP 1** and CUR are comparatively closer. Taking into account commonly used reference antioxidants (such as gallic acid, ascorbic acid, and butylated hydroxytoluene), they generally exhibit DPPH IC_50_ values in the range of 0.5–5.0, 2–10, and 10–30 μg mL^−1^, respectively, while ABTS IC_50_ values are typically reported in the ranges of 1–5, 2–8, and 5–20 μg mL^−1^, depending on the experimental conditions employed [23,24]. These literature data indicate that compound **CUR-CO-PP 2** displays antioxidant activity comparable to that of the most efficient antioxidants, particularly ascorbic acid and gallic acid, whereas CUR and **CUR-C3-PP 1** remain within the range of highly active radical scavengers. In the FRAP assay, which evaluates the reducing power of the tested compounds, solutions of 1.60 ± 0.14 μg mL^−1^ of compound **CUR-CO-PP 2**, 1.80 ± 0.16 μg mL^−1^ of compound CUR, and 1.82 ± 0.11 μg mL^−1^ of compound **CUR-C3-PP 1** produced a reducing capacity equivalent to 1.0 μg mL^−1^ Trolox. The lower concentration required for compound **CUR-CO-PP 2** to achieve the same Trolox-equivalent response further confirms its superior antioxidant potential. On the other hand, compounds **CUR-C3-PP 1** and CUR displayed very similar ferric-reducing capacities, suggesting only minor differences in their electron-donating ability. Moreover, the **CUR-CO-PP 2** concentration (1.60 ± 0.14 μg mL^−1^), required to achieve a Trolox-equivalent response highlights its remarkable reducing capacity and places its antioxidant performance within the range of highly active phenolic antioxidants [25,26].

### 2.3. In Silico Studies

**Figure 3:**
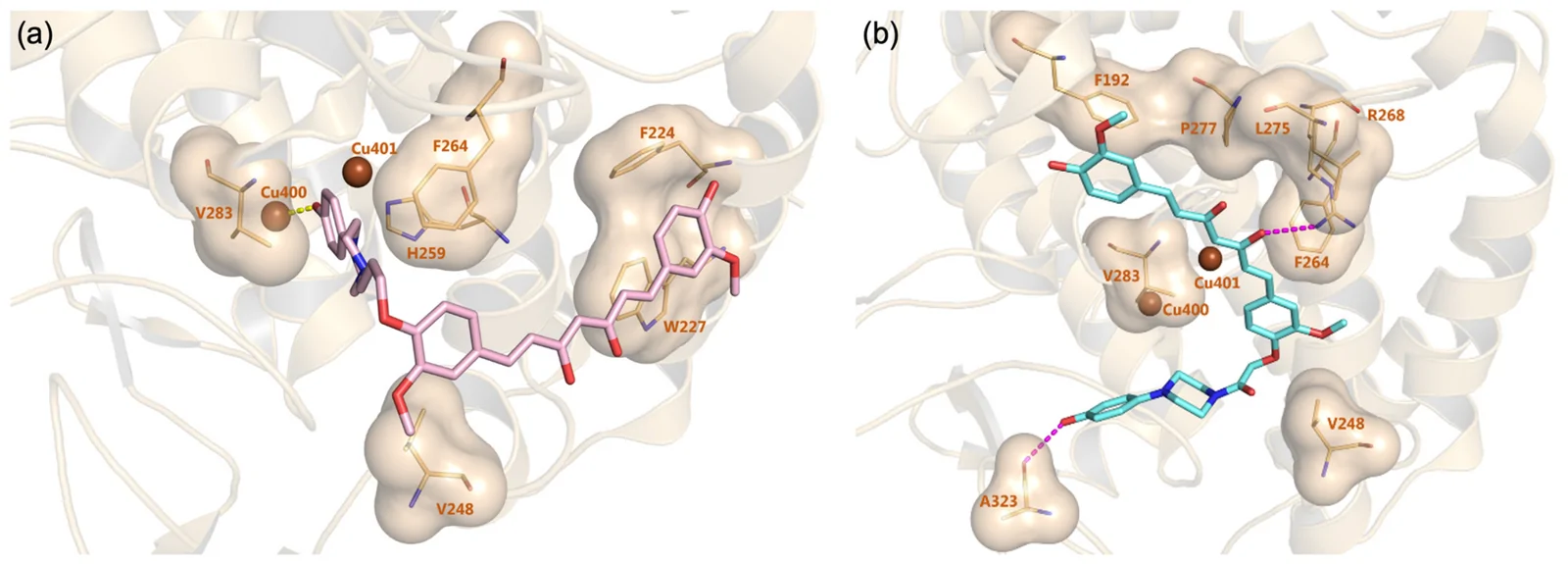
Three-dimensional representation of the molecular docking studies of **CUR-C3-PP 1** (panel (**a**)) and **CUR-CO-PP 2** (panel (**b**)) within the *Ab*TYR active site (RCSB PDB: 2Y9X) [25]. The protein backbone is depicted as a wheat cartoon structure, while copper ions are represented as brown spheres. Amino-acidic residues involved in hydrophobic interaction are displayed as surface representations, while those participating in other protein–ligand interactions are shown as sticks. The ligands are represented as sticks coloured in light pink (**CUR-C3-PP 1**, (**a**)) and aquamarine (**CUR-CO-PP 2**, (**b**)). Coordination bonding interactions are depicted as yellow dashed lines. The image was generated using PyMOL v.3.1.4.1.

To corroborate the experimental kinetic data and elucidate the molecular basis underlying the different inhibition mechanisms of curcuminoids **CUR-C3-PP 1** and **CUR-CO-PP 2**, molecular docking studies were carried out. The crystal structure of tyrosinase from *Ab*TYR was retrieved from the Protein Data Bank (PDB ID: 2Y9X) and used for all docking calculations [27]. In more detail, the binding modes of the two synthesized CUR derivatives (**CUR-C3-PP 1** and **CUR-CO-PP 2**) were predicted using the same validated docking protocol previously described in our recent studies [12,22,28]. Docking analysis revealed that both derivatives established key interactions likely responsible for their inhibitory activity toward *Ab*TYR (Figure 3a,b).

The inhibitor **CUR-C3-PP 1** was predicted to successfully access the catalytic cavity, adopting a favorable binding conformation within the active site (see Figure 3a). As expected, its 4-(piperazin-1-yl)phenol moiety extended into the catalytic pocket interacting with the Cu400 ion through the hydroxy-phenolic group. This orientation was consistent with the binding mode that has already been observed for our previously reported compounds possessing this pharmacophoric feature [12]. In addition, the aromatic ring of this moiety engaged a π–π stacking interaction with H259, further contributing to ligand binding stabilization within the catalytic site of *Ab*TYR. The ligand−protein complex was also reinforced by multiple hydrophobic interactions with residues F224, W227, V248, F264, and V283. Overall, these interactions were consistent with the proposed mechanism of action of **CUR-C3-PP 1** as an inhibitor of TYR. The docking analysis identified the competitive pose as the most likely binding mode, in line with the predominant, though not exclusive, competitive component of the mixed-type inhibition mechanism diagnosed kinetically.

For compound **CUR-CO-PP 2**, the docking studies suggested that this inhibitor was still able to fill the *Ab*TYR cavity (Figure 3b) but compared with **CUR-C3-PP 1**, the replacement of the flexible three-carbon linker with an amide-containing linker resulted in a markedly different binding orientation within the catalytic cavity. In detail, it did not establish interactions with the catalytic copper ions; the absence of direct interactions with the catalytic copper ions was consistent with its experimentally observed non-competitive inhibition of *Ab*TYR. Instead, the complex was stabilized through an extensive network of hydrophobic interactions with residues F192, V248, F264, L275, P277, V283, and A323, together with a π–π stacking interaction with F192, which further contributed to complex stabilization. Two hydrogen bond interactions were also identified: the first one involved the oxygen atom of A323 residue and the hydroxyphenolic group of the 4-(piperazin-1-yl)phenol moiety; whereas the second hydrogen bond was formed between the hydroxyl group of the curcumin scaffold and the amino group of R268. Considering the noncompetitive mechanism of **CUR-CO-PP 2**, we performed a blind docking analysis beyond the canonical copper-binding site. Under the employed docking conditions, no alternative binding site with a distinct binding mode was identified for **CUR-CO-PP 2**, consistent with the results shown in Figure 3b.

Subsequently, docking scores and binding free energies were calculated for the docking position of the two studied compounds **CUR-C3-PP 1** and **CUR-CO-PP 2**. The docking scores of the best-ranked poses identified by the program GOLD [29] were 47.47 for **CUR-C3-PP 1** and 39.24 for **CUR-CO-PP 2**; while binding free energies, calculated using the molecular mechanics/generalized Born surface area (MM-GBSA) method implemented in Prime from the Schrödinger suite [30], yielded ΔG binding values of −74.01 and −61.84 for **CUR-C3-PP 1** and **CUR-CO-PP 2**, respectively. Therefore, these calculations confirmed a good correlation between the computational and experimental data. This good correlation was further confirmed by the docking scores and binding free energies calculated for the reference compounds **PP** and CUR, that were reported for a comparative purpose in the Supplementary Materials.

Finally, in order to further investigate the various tautomeric and ionic forms, the binding modes of the diketo tautomer of each new compound and the relative enolate species were also examined and are discussed in the Supplementary Materials, revealing a binding mode slightly different from that of the enolic form, yet still consistent with the proposed mechanism of action (see Figures S1–S4).

### 2.4. Cellular Fluorescence Imaging Studies

To investigate the suitability of the synthesized curcumin derivatives as imaging agents, **Cur-C3-PP 1** and **Cur-CO-PP 2** were evaluated in B16-F10 murine melanoma cells and compared with two non-melanogenic cell lines, NIH-3T3 mouse fibroblasts and MDA-MB-231 human breast cancer cells. To evaluate in vitro models with varying levels of TYR, the murine melanoma cell line B16-F10 was selected for the current phase due to its high tyrosinase expression. This was utilized alongside the murine NIH-3T3 fibroblast and human MDA-MB-231 cell lines, both lacking in tyrosinase expression. Fluorescence microscopy analyses were performed under identical experimental conditions to compare the intracellular emission and localization of both derivatives.

**Figure 4:**
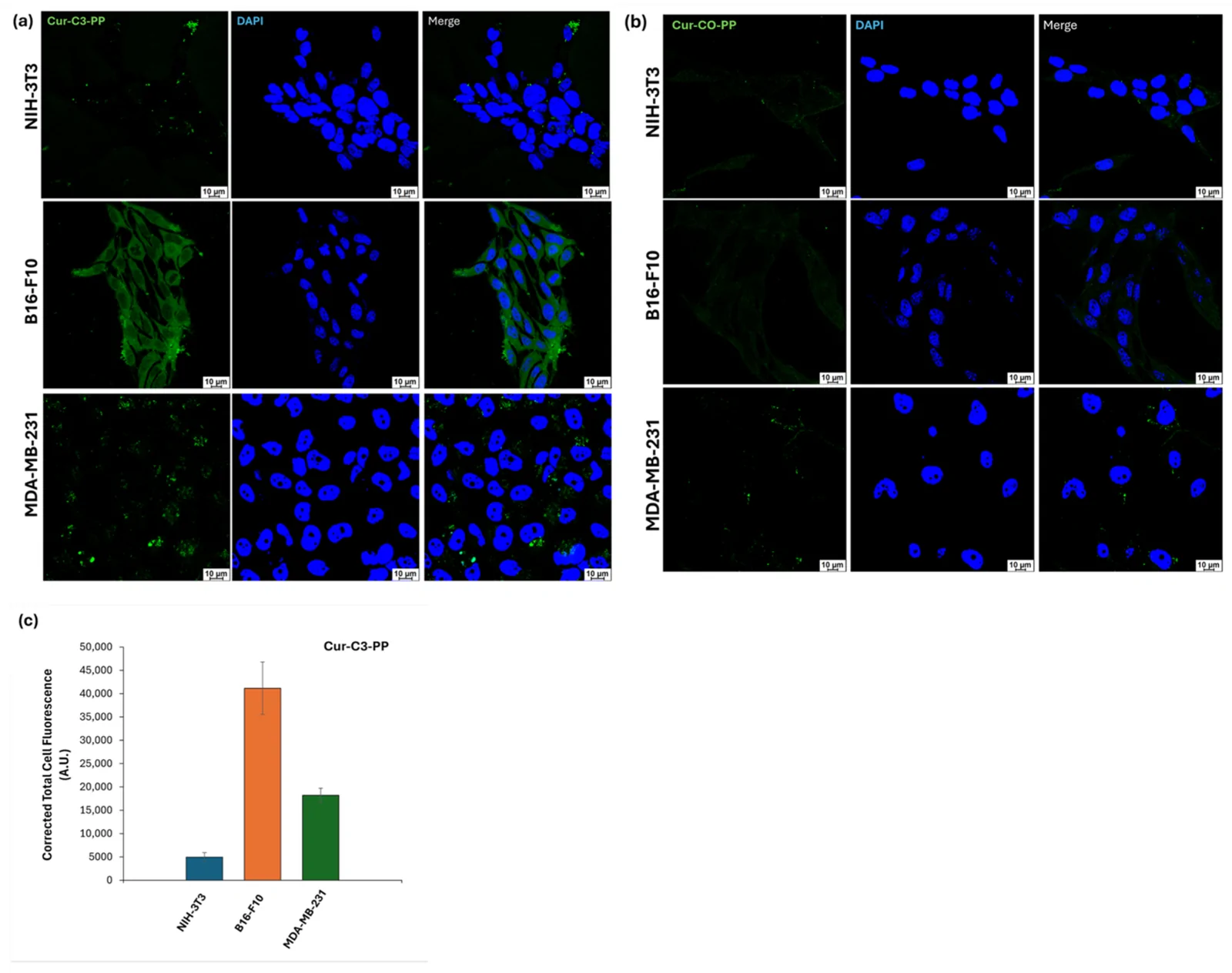
Fluorescence microscopy images of NIH-3T3 mouse fibroblasts, B16-F10 murine melanoma, and MDA-MB-231 human triple-negative breast cancer cells treated with 20 μM of (**a**) **CUR-C3-PP 1,** (**b**) **CUR-CO-PP 2,** and (**c**) quantitative fluorescence analysis of 20 μM of **Cur-C3-PP 1** in different cell lines expressed as Corrected Total Cell Fluorescence (CTCF) in arbitrary units (A.U.). Data are presented as mean ± SEM.

**Figure 5:**
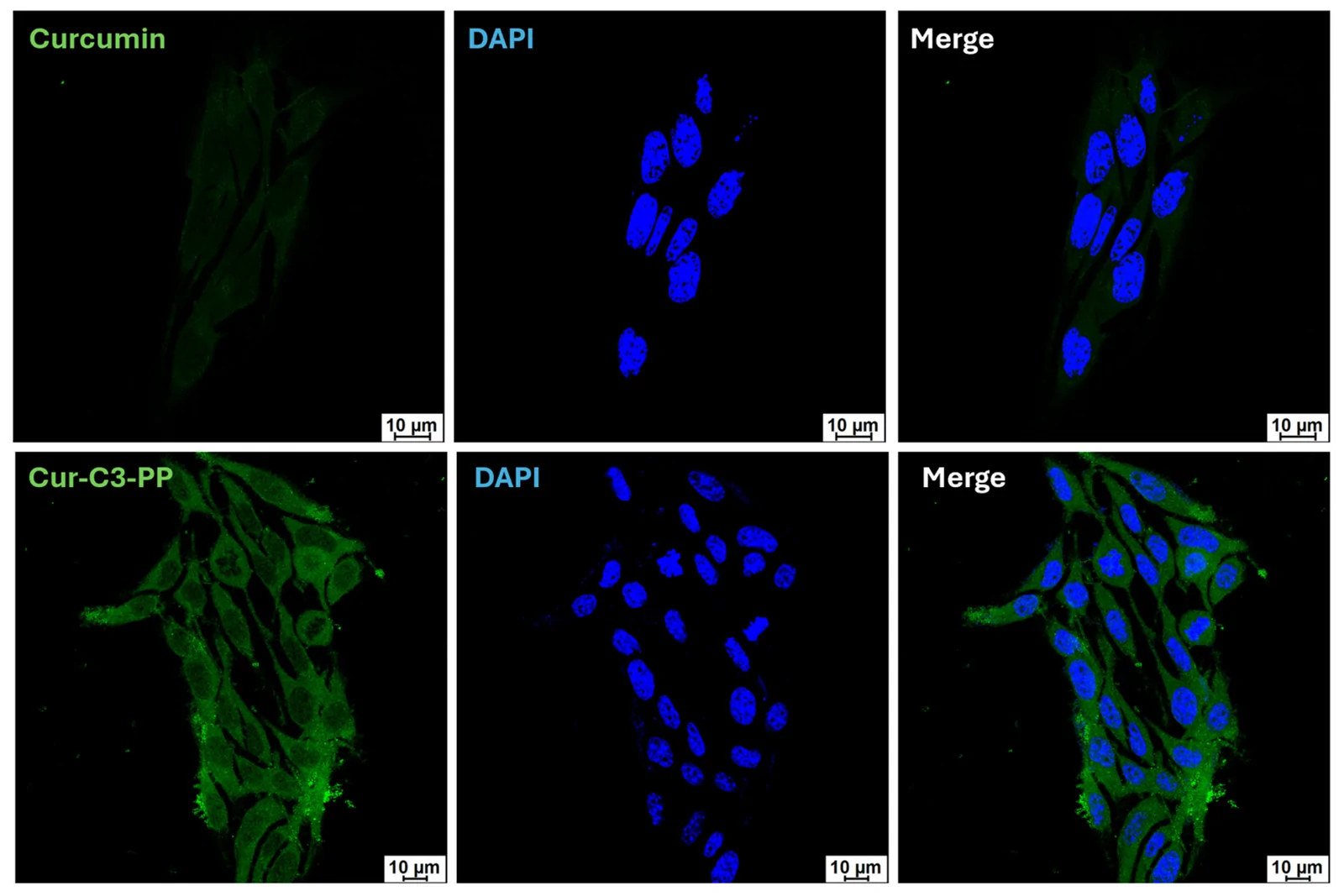
Fluorescence microscopy images of B16-F10 murine melanoma cell line treated with 20 μM of CUR or **CUR-C3-PP 1**.

**CUR-C3-PP 1** exhibited a strong intracellular fluorescence exclusively in B16-F10 murine melanoma cells, as shown in Figure 4a, whereas no appreciable emission was detected in NIH-3T3 fibroblasts or MDA-MB-231 breast cancer cells. Quantitative fluorescence intensity per cell was measured for each cellular line and is shown in Figure 4 (panel c). Interestingly, control experiments performed with CUR alone (at the same concentration) in B16-F10 murine melanoma cells (Figure 5, compared to **CUR-C3-PP 1**) did not reveal any significant intracellular fluorescence under the same experimental conditions, suggesting that the parent fluorophore CUR is either poorly internalized or does not accumulate to detectable levels within these cells. Time- and dose-dependent uptake experiments were performed for CUR-C3-PP 1 in B16-F10 murine melanoma cells (Figures S18 and S19, Supplementary Materials). These experiments demonstrated that a relevant fluorescence signal was already detectable at a concentration of 5 μM with the maximum observed at 4 h. This observation may be associated with preferential accumulation of the compound within melanogenic compartments and/or with a restriction of intramolecular motions upon cellular uptake, which could contribute to enhanced fluorescence emission.

**CUR-CO-PP 2** exhibited a negligible fluorescence (Figure 4b) in the investigated cell lines, that cannot be simply attributed to poor cellular uptake, since the compound clearly reaches its intracellular target and retains high biological activity, as early documented by its stronger depigmenting activity (vide infra) **CUR-C3-PP 1**. A plausible explanation is that the higher affinity of **CUR-CO-PP 2** for tyrosinase promotes its preferential association with the enzyme or with melanogenic compartments, where the local biochemical environment may influence its emissive properties. Alternatively, differences in intracellular localization or microenvironment, rather than intrinsic structural features alone, may account for the markedly weaker fluorescence observed for compound **CUR-CO-PP 2**.

### 2.5. Cytotoxicity

**Figure 6:**
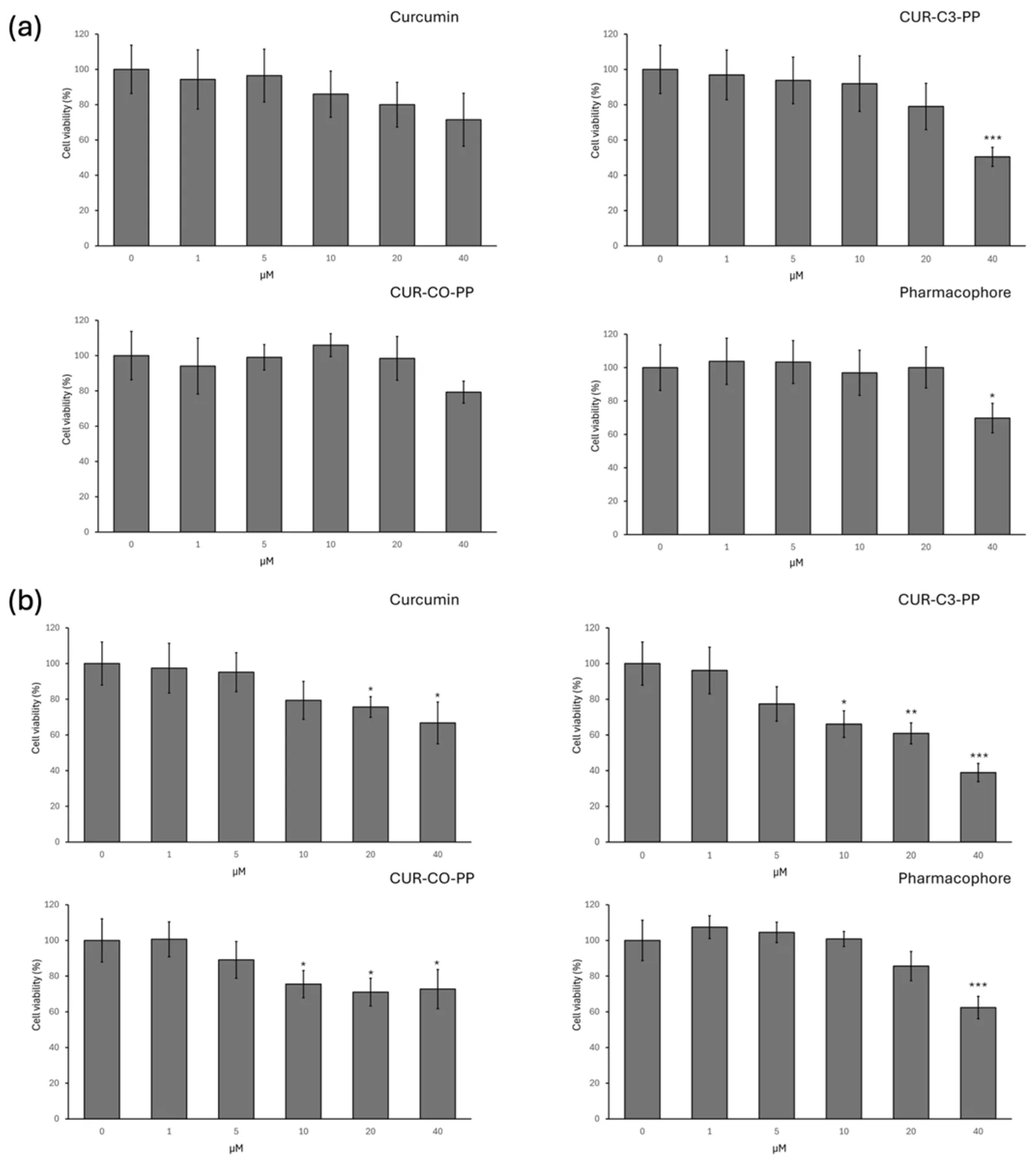
Cell viability of (**a**) NIH-3T3 fibroblasts, and (**b**) B16-F10 melanoma cells following 24 h treatment with CUR, **CUR-C3-PP 1**, **CUR-CO-PP 2,** and **PP**. Cell viability was determined after exposure to increasing concentrations of each compound (1–40 μM). Data are shown as mean ± SD. Asterisks indicate statistically significant differences compared to the control (* *p* < 0.05, ** *p* < 0.01, *** *p* < 0.001, Student’s *t*-test).

The cytotoxicity of **CUR-C3-PP 1** and **CUR-CO-PP 2** was evaluated after 24 h of incubation in NIH-3T3 mouse fibroblasts and B16 murine melanoma cells and compared with that of CUR and **PP**. Overall, all compounds exhibited good biocompatibility at concentrations up to 20 μM, with cell viability generally remaining above 70–80% in both cell lines (Figure 6a,b). A more pronounced reduction in viability was observed only at the highest concentration tested (40 μM). Interestingly, the two conjugates **CUR-C3-PP 1** and **CUR-CO-PP 2** displayed distinct cytotoxicity profiles in the B16-F10 cell line (Figure 6b). Compound **CUR-C3-PP 1** induced a concentration-dependent decrease in B16-F10 cell viability (Figure 6b), which was more pronounced than that observed in NIH-3T3 cells cells (Figure 6a), suggesting a certain degree of preferential activity toward melanoma cells. In contrast, **CUR-CO-PP 2** exhibited a milder cytotoxic effect, maintaining higher cell viability in both cell lines over the entire concentration range. Notably, in B16-F10 cells, its cytotoxicity remained comparable to, or lower than that of the parent pharmacophore while preserving its superior anti-melanogenic activity. Conversely, the greater reduction in B16-F10 viability observed for **CUR-C3-PP 1** may reflect its preferential interaction with melanoma cells, in agreement with its selective intracellular fluorescence and its potential application as a melanoma-targeted imaging probe.

### 2.6. In Vivo Zebrafish Assay

Zebrafish (*Danio rerio*) embryos have emerged as a powerful, phenotype-based tool for the rapid in vivo screening of anti-melanogenic compounds [31]. Due to their optical transparency, high skin permeability, and physiological conservation with humans, they offer a reliable bridge between preliminary in vitro assays and complex mammalian models.

**Figure 7:**
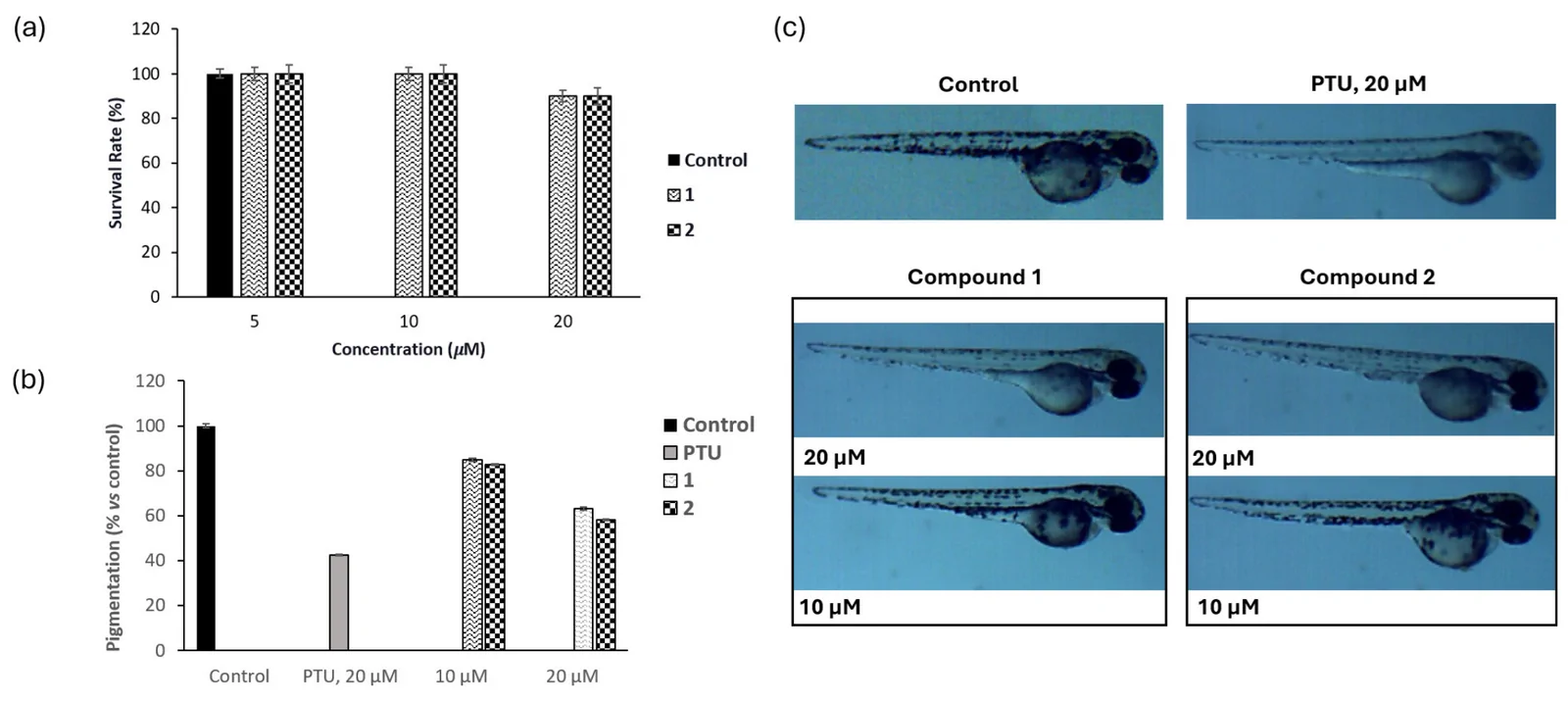
(**a**) Survival rate of 72 hpf zebrafish embryos after treatments; (**b**) Effects of tested compounds (**CUR-C3-PP 1**, **CUR-CO-PP 2,** and **PTU**) on the pigmentation of 72 hpf zebrafish embryos expressed as % vs. control; (**c**) Lateral view of 72 hpf zebrafish. Images were captured using a stereomicroscope (SMZ-171 Series, Motic) equipped with a digital camera (Moticam^®^ 5 plus, Motic, San Antonio, TX, USA).

To assess in vivo biocompatibility and efficacy, zebrafish embryos at 24 h post-fertilization (hpf) were treated with compounds **CUR-C3-PP 1** and **CUR-CO-PP 2**. Notably, no acute toxicity phenotypes were detected, and a high survival rate was maintained upon exposure to concentrations up to 20 μM (Figure 7a). These findings represented a significant improvement over the parent compound CUR, as previous studies highlighted its acute toxicity in zebrafish embryos at concentrations exceeding 5 μM [15].

Based on these safety profiles, the depigmenting efficacy of both CUR- derivatives was evaluated within a 5–20 μM concentration range. Following a 48 h incubation, melanin pigmentation was assessed via stereomicroscopy, and the captured images were quantified using ImageJ software (version 1.54g). Quantitative data, expressed as a percentage of pigmentation relative to the vehicle control (1% DMSO, set at 100%), are summarized in Figure 7b. Both derivatives **CUR-C3-PP 1** and **CUR-CO-PP 2** elicited a concentration-dependent reduction in embryo pigmentation. Specifically, the highest tested concentration (20 µM) led to residual pigmentation levels of 63.1% for **CUR-C3-PP 1** and 58.2% for **CUR-CO-PP 2**. Compound **CUR-CO-PP 2** exhibited a pronounced depigmenting effect, approaching the marked inhibition observed for the positive control 1-phenyl-2-thiourea (PTU), which reduced pigmentation to 42.6% at the same dose (Figure 7c). A 10 µM concentration induced only a mild decrease in pigmentation (residual levels of 84.9% and 82.8%, respectively), whereas the lowest dose (5 µM) exerted no observable depigmenting effect.

## 3. Materials and Methods

### 3.1. Chemicals and Instruments

**Chemicals**. All Chemicals and solvents were purchased from Merck-Italy and, when necessary, purified according to standard procedures. Curcumin was purchased from Merck with purity ≥ 65% and was further purified by column chromatography using silica gel as stationary phase and CHCl_3_/*n-*hexane 90/10. All the syntheses were monitored by TLC on commercially available precoated plates (silica gel 60 F_254_), and the products were visualized with vanillin [1 g dissolved in MeOH (60 mL) and conc. H_2_SO_4_ (0.6 mL)] under UV lamp illumination. Silica gel 60 was used for column chromatography. Tyrosinase lyophilized powder, ≥1000 unit/mg solid was purchased from Merck and used without purification.

**Instruments.** Proton (^1^H) and carbon (^13^C) NMR spectra were recorded on a Varian 500 spectrometer at 500 MHz for ^1^H and 125 MHz for ^13^C using CDCl_3_, DMSO-d_6_. Chemical shifts are given in parts per million (ppm) (δ) relative to CDCl_3_ δ 7.26 ppm for proton and 77.16 ppm for carbon, relative to DMSO-d_6_ δ 2.5 ppm for proton and 39.52 ppm for carbon. Coupling constants (J) are given in hertz, and the attributions are supported by heteronuclear single-quantum coherence (HSQC) and correlation spectroscopy (COSY) experiments. For clarity, the assignment of the ^1^H NMR signals should be interpreted with reference to the corresponding molecular structures reported in the Supplementary Materials. Elemental analysis was performed with Eurovector EA3100 CHNS Elemental Analyzers (EuroVector S.r.l., Pavia, Italy). Melting points (mp) were determined with Buchi B-545 apparatus (BUCHI Labortechnik AG Flawil, Switzerland) and are uncorrected. A stereomicroscope (SMZ-171 Series, Motic, Hong Kong, China) equipped with a digital camera (Moticam^®^ 5 plus, Motic, San Antonio, TX, USA) was used for image acquisition. Luminescence was recorded using a GloMax luminometer (Promega, Madison, WI, USA). Leica Stellaris 5 confocal microscope (Leica Microsystems, Wetzlar, Germany) equipped with a 63× oil immersion objective.

### 3.2. Chemistry

#### 3.2.1. Synthesis of Curcumin Derivative **CUR-C3-PP 1**

Compound **4** (100 mg, 0.20 mmol), 4-(piperazin-1-yl)phenol (**PP**) (44 mg, 0.24 mmol) and NaHCO_3_ (20 mg, 0,24 mmol) were dissolved in 12 mL of dry CH_3_CN and stirred, under inert atmosphere, at overnight reflux. The solvent was removed and the crude was purified by column chromatography (SiO_2_; CH_3_Cl/MeOH 9:1) to give compound **CUR-C3-PP 1** (64 mg, 58%) as yellow solid. R_f_ = 0.33 eluant CHCl_3_/MeOH 9/1; mp 122–124 °C; ^1^H NMR (500 MHz, DMSO-*d_6_*) δ: 7.56 and 7.55 (2x d, *J* = 15.5 Hz, 2x 1H, H-4 and H-4′); 7.34 and 7.32 (2x d, *J* = 2.0 Hz, 2x 1H, H-6 and H-6′); 7.24 and 7.15 (2x dd; *J* = 8.5 Hz, *J* = 2.0 Hz, 2x 1H, H-10 and H-10′); 7.02 (d, *J* = 8.5 Hz, 1H, H-9′); 6.86–6.71 (m, 3H, H-9, H-3, H-3′); 6.77 and 6.64 (AA’BB’ system, *J* = 8.9 Hz, 4H, H_ar_); 6.08 (s, 1H, H-1); 4.07 (t, *J* = 6.4 Hz, 2H, H-α); 3.84 (s, 6H, 2x -OMe); 3.02–2.88 (m, 4H, H_pip_); 2.57–2.37 (m, 6H, H_pip_ and H-γ);1.91 (m, 2H, H-β). ^13^C NMR (126 MHz, DMSO-*d_6_*) δ:184.1; 183.1; 151.3; 150.7; 150.0 149.6; 148.5; 144.7; 141.4; 140.6; 128.0; 126.6; 123.7; 123.3; 122.5; 121.5; 118.1; 116.2; 115.9; 113.2; 111.8; 111.1; 101.4; 67.1; 56.11; 54.8; 55.3; 50.4; 26.2. Anal. For (C_34_H_38_N_2_O_7_) C 69.61, H 6.53, N 4.77, O, 19.09, Found: C 69.58; H 6.56, N 4.73.

#### 3.2.2. Synthesis of Curcumin Derivative **CUR-CO-PP 2**

Curcumin (100 mg, 0.27 mmol), compound **7** (69 mg, 0.27 mmol) and K_2_CO_3_ (75 mg, 0.54 mmol) were dissolved in dry DMF (10 mL), and the mixture was stirred, under inert atmosphere at 80 °C overnight. The solvent was removed under reduced pressure, and the crude was dissolved in CHCl_3_ and washed with water (3 × 50 mL), the organic layers were collected, dried over anhydrous Na_2_SO_4_, filtered and the solvent was removed under vacuum. The crude was purified by column chromatography (SiO_2_, CH_2_Cl_2_/MeOH 93:7 to 9:1) affording the desired compound **CUR-CO-PP 2** (R_f_ = 0.30, CH_2_Cl_2_/MeOH 96:4; mp 114–116 °C; 32 mg, 20%) as yellow solid. ^1^H NMR (500 MHz, CDCl_3_) δ: 7.59 and 7.58 (2x d, *J* = 15.5 Hz, 2H, H-3 and H-3′); 7.15–7.09 (m, 2H, H-10 and H-10′); 7.08 and 7.05 (2x d, *J* = 2.0 Hz, 2H, H-6 and H-6′); 6.97 and 6.93 (2x d, *J* = 8.0 Hz, 2H, H-9 and H-9′); 6.83 and 6.78 (AA’BB’ system, 4H, H_ar_); 6.49 and 6.48 (2x d, *J* = 15.5 Hz, 2H, H-4 and H-4′); 5.88 and 5.82 (2x s, 2H, H-1 and OH); 4.83 (s, 2H, H-11); 3.95 and 3.92 (2x s, 6H, -OMe); 3.83–3.70 (m, 4H H_pip_) and 3,09–2.97 (m, 4H, H_pip_). ^13^C NMR (126 MHz, CDCl_3_) δ: 183.9, 182.9, 166.3, 150.5, 149.8, 149.8, 148.1, 147.0, 145.4, 140.9, 140.1, 129.6, 127.8, 123.1, 122.8, 122.3, 121.9, 119.3, 116.1, 115.0, 113.9, 110.8, 109.8, 110.2, 101.4, 68.8, 56.1, 52.6, 52.0, 45.6, 42.5. Anal. For (C_33_H_34_N_2_O_8_) C 67.56, H 5.84, N 4.78, O 21.82. Found: C 67.53, H 5.76, N 4.81.

#### 3.2.3. Photophysical Characterization of Derivatives **CUR-C3-PP 1** and **CUR-CO-PP 2** vs. CUR

Electronic absorption spectra were acquired with a Jasco V-560 UV–Vis spectrophotometer, while steady-state fluorescence spectra were collected using a HORIBA Jobin Yvon Fluorolog-3 spectrofluorimeter (Kyoto, JAPAN) equipped with a Hamamatsu R928P photomultiplier tube.

### 3.3. Biology

#### 3.3.1. Tyrosinase Inhibition Assay and Kinetic Mechanism Analysis

According to the previously reported method [32] the tyrosinase inhibition assay was carried out with minor modifications. Aliquots (0.05 mL) of compounds **CUR-C3-PP 1**, **CUR-CO-PP 2,** CUR and KA dissolved in DMSO (10–200 μM), were mixed with 0.9 mL of PBS buffer (pH 6.8). Then, 0.05 mL of the *Ab*TYR (EC 1.14.18.1, purchased by “Merck Life Science S.r.l.”) was combined to the mixture, which was incubated (10 min) at 25 °C. Finally, 0.5 mL of substrate solution (L-Tyrosine or L-DOPA, 1.25 mM) was added. After incubation of 30 and 10 min for monophenolase or diphenolase activity, respectively, absorbance was recorded at 475 nm employing a spectrophotometer (Agilent Technologies, Cary 60, UV/Vis). The experiments were performed in triplicate, and the results are given as mean of concentrations leading to half-maximal response (IC_50_) ± standard deviation (SD). Following the same procedure (volumes and incubations), further experiments were performed to determine the mechanism of inhibition of compounds **CUR-C3-PP 1** and **CUR-CO-PP 2**. Three different concentrations (14.20–28.40–56.80 μM and 7.10–14.20–28.40 μM for compounds **CUR-C3-PP 1** and **CUR-CO-PP 2**, respectively) were used for kinetic studies. The reaction mixture consisted of *Ab*TYR mixed with four concentrations of the substrate L-DOPA (0.6–5 mM), in phosphate buffer (pH 6.8). The results obtained were used to build distinct Lineweaver−Burk plots for each compound.

#### 3.3.2. Antioxidant Assay

*DPPH Radical Scavenging Assay:* The assay was carried out following a previously reported procedure [33]. Briefly, an aliquot of each compound was mixed with 950 μL of an 80 μM ethanolic DPPH^•^ solution, to assess antioxidant activity over the concentration range of 0.0–50.0 μg/mL, and incubated for 30 min. The absorbance was then measured at 517 nm. The IC_50_ value represents the sample concentration required to reduce the absorbance at 517 nm by 50%.

*ABTS Radical Scavenging Assay:* The assay was performed according to the method reported by Re et al. [23]. The ABTS^•+^ radical cation was generated by persulfate oxidation of ABTS. Briefly, ABTS (7.0 mM) was mixed with ammonium persulfate (10 mM) and kept overnight in the dark at room temperature for 12–16 h before use. The resulting solution was diluted with PBS until its absorbance at 734 nm reached approximately 1.0. An aliquot of each sample was then added to 1.0 mL of the ABTS^•+^ solution to assess antioxidant activity over the concentration range of 0.0–50.0 μg/mL, and the decrease in absorbance was recorded at 734 nm after 6 min. The IC_50_ value represents the sample concentration required to reduce the absorbance at 734 nm by 50%.

*Ferric-Reducing Antioxidant Power (FRAP) Assay:* The assay was performed according to the method described by Benzie and Strain [34]. The FRAP reagent was freshly prepared using 300 mM acetate buffer at pH 3.6, 10 mM 2,4,6-tripyridyl-s-triazine (TPTZ) dissolved in 40 mM HCl, and 20 mM FeCl_3_·6H_2_O. These three solutions were mixed at a 10:1:1 (*v*/*v*/*v*) ratio. An aliquot of 25.0 μL of each tested compound was added to 0.750 mL of FRAP reagent to assess antioxidant activity over the concentration range of 0.0–50.0 μg/mL. After incubation at 37 °C for 4 min, the absorbance of the reaction mixture was measured at 593 nm. All measurements were performed in triplicate. Ferric-reducing antioxidant power was calculated from a linear calibration curve and expressed as Trolox equivalents.

### 3.4. In Silico Studies

Ligand preparation was performed using Software VegaZZ v 3.2.4 [35]. Partial charges were calculated according to the Gasteiger method under the CHARMM force field. Subsequently, energy minimization and conformational searches were executed utilizing the integrated AMMP (Automated Molecular Mechanics Prediction) engine. During the conformational search, the selected rotatable bonds were defined as flexible torsions to allow the exploration of different molecular conformations. The prepared ligands were docked into the crystal structure of *Ab*TYR (PDB code: 2Y9X) [27] using GOLD software (v. 2026) [29] following a protocol developed and validated by our research team [22,28]. The ASP (Astex Statistical Potential) scoring function was employed for pose evaluation, and the binding site was delineated using a grid box centered at coordinates x = −10.021, y = −28.8230, and z = −43.5960. For each ligand, one hundred docking poses were generated, and pose clustering was performed with an RMSD threshold of 0.8 Å. The “Allow early termination” option was deactivated to guarantee comprehensive conformational sampling. For each ligand, the highest-scoring docking pose was retained for subsequent analysis.

### 3.5. Fluorescence Imaging Studies and Cytotoxicity Studies

#### 3.5.1. Cell Culture

The murine B16-F10 melanoma, murine NIH-3T3 fibroblast, and human MDA-MB-231 cell lines were cultured in Dulbecco’s Modified Eagle Medium (DMEM) supplemented with 10% fetal bovine serum (FBS), 100 U/mL penicillin, and 100 μg/mL streptomycin. Cells were grown and maintained according to standard cell culture protocols and kept in a humidified atmosphere with 5% CO_2_ at 37 °C. Upon reaching subconfluence, cells were either passaged or seeded for subsequent experimental procedures. Mycoplasma screening of cultured cell lines was routinely performed using the Mycoplasma PCR Detection Kit (Cat. No. G238 Applied Biological Materials Inc., Richmond, BC, Canada) according to the manufacturer’s instructions.

#### 3.5.2. Cell Viability Assay

Cell viability was evaluated using the CellTiter-Glo Luminescent Cell Viability Assay kit (Promega, Madison, WI, USA) according to the manufacturer’s instructions, which quantifies ATP bioluminescence as an indicator of metabolically active cells. Briefly, B16-F10 or NIH-3T3 cells were seeded into opaque-walled 96-well cell culture plates at the density of 2500–5000 cells/well in 100 μL of complete medium and allowed to adhere overnight in a humidified atmosphere at 37 °C with 5% CO_2_. The following day, cells were exposed to the indicated concentrations (1, 5, 10, 20, and 40 μM) of curcumin, **CUR-C3-PP 1** and **CUR-CO-PP 2**, or the pharmacophore core, followed by a 24 h incubation. Post-treatment, the plates were equilibrated to room temperature for approximately 30 min. A volume of CellTiter-Glo reagent equivalent to the culture medium volume (100 μL) was added to each well. The plates were agitated on an orbital shaker for 2 min to ensure complete cell lysis and then incubated for 10 min at room temperature in the dark to stabilize the luminescent signal. Luminescence was recorded using a GloMax luminometer (Promega, Madison, WI, USA). Relative cell viability (%) was calculated by normalizing the luminescent intensity of the treated groups to that of the vehicle-treated control wells (designated as 100%). All experiments were performed in at least three biological replicates (n=3). Data are expressed as mean ± standard deviation (SD).

#### 3.5.3. Fluorescence Microscopy Analysis

For intracellular uptake experiments, murine B16-F10 melanoma, murine NIH-3T3 fibroblast, and human MDA-MB-231 triple-negative breast cancer cells were seeded onto glass coverslips. Following a 24 h incubation period, the cells were subsequently treated with varying concentrations of curcumin, **CUR-C3-PP 1** and **CUR-CO-PP 2** compounds. Following an additional 24 h incubation, cells were fixed in 4% paraformaldehyde for 15 min at room temperature. Nuclei were counterstained with 0.5 μg/mL of 4′,6-diamidino-2-phenylindole dihydrochloride (DAPI; Sigma-Aldrich, St. Louis, MO, USA). The coverslips were rinsed, recovered, and mounted onto glass slides using 10 μL of ProLong Diamond Antifade Mountant (Invitrogen, Thermo Fisher Scientific, Waltham, MA, USA). High-resolution images were acquired using a Leica Stellaris 5 confocal microscope (Leica Microsystems, Wetzlar, Germany) equipped with a 63× oil immersion objective. Fluorescence intensity per cell was quantified using ImageJ/Fiji software (version 1.54g, Java 1.8.0_345, 64-bit; NIH, Bethesda, MD, USA) (according to standard protocols. Corrected Total Cell Fluorescence (CTCF) was calculated using the formula: CTCF = Integrated Density—(Area of selected cell × Mean background fluorescence). Data were plotted as mean ± SEM of single-cell measurements across three independent biological replicates.

### 3.6. In Vivo Zebrafish Assay

Adult male and female *Danio rerio* (wild-type AB strain) were kept in a temperature-controlled aquarium (28.5 °C) under a 14/10 h light/dark photoperiod and fed with live *Artemia salina*. The eggs obtained from natural spawning were collected and incubated at 28.5 °C. To exclude damaged or unfertilized eggs, they were examined under a stereomicroscope. After 24 h, embryos exhibiting normal cleavage and developmental synchronization were selected to ensure experimental homogeneity. The vitality and whitening effect were evaluated on zebrafish embryos based on literature procedure [36]. The eggs at 24 hpf (hours post-fertilization) were manually stripped of the chorion, and the embryos were distributed into 96-well plates (1 embryo per well), randomly divided into experimental groups. Embryos were treated with compounds **CUR-C3-PP 1** and **CUR-CO-PP 2** (5–20 μM), previously solubilized in DMSO (1%). Negative controls were incubated with DMSO (1%), while positive controls with PTU (20 μM). The sample size was set at n = 10 embryos per group, replicated in independent experiments (N = 3), determined to be sufficient for statistical power based on previous laboratory data. After 48 h of incubation at 28 °C (72 hpf), the effects of the treatment were observed using stereomicroscope (SMZ-171 Series, Motic, Hong Kong, China) equipped with a digital camera (Moticam^®^ 5 plus) for image acquisition. The mortality for each treatment group was recorded. “ImageJ” was employed to evaluate the pigmentation rate. Images were converted to grey scale and the pixels corresponding to the pigmentation rate were quantified. The pigmentation rate was expressed as percentage compared to the untreated embryos (100%). To minimize observer bias, measurements were carried out by an investigator blinded to the treatment allocation. Data were analyzed by one-way analysis of variance (ANOVA). Values of *p* < 0.05 and *p* < 0.01 were regarded as indicating a significant difference. All experiments involving zebrafish embryos < 120 hpf were not subject to regulations according to the European Directive 2010/63/EU and they were performed following the ethical guidelines described by the National Institute of Health Guide for the Care and Use of Laboratory Animals.

## 4. Conclusions

In summary, two novel curcumin derivatives, **CUR-C3-PP 1** and **CUR-CO-PP 2**, were successfully synthesized, one using an optimized convergent strategy based on an adapted Pabon methodology significantly improving the yield of compound **CUR-C3-PP 1**, the other by the direct functionalization of the parent CUR to achieve compound **CUR-CO-PP 2** in acceptable yield.

The structural modifications engineered in molecules **CUR-C3-PP 1** and **CUR-CO-PP 2** successfully amplified their tyrosinase inhibitory capacity compared to the parent lead, CUR. Among the series, derivative **CUR-CO-PP 2** stands out as the most promising candidate, significantly eclipsing the potency of CUR and exceeding the standard inhibitor KA against both the monophenolase and diphenolase activities of *Ab*TYR. Kinetic and molecular docking analyses successfully rationalized this enhanced profile, demonstrating that the structural transition from a flexible aliphatic chain in **CUR-C3-PP 1** to an amide-bearing linker in **CUR-CO-PP 2** critically dictates their divergent binding modes and inhibition mechanisms within the catalytic cavity.

In contrast to CUR, whose tyrosinase inhibitory effects are often mediated by its robust antioxidant properties, the engineered derivatives **CUR-C3-PP 1** and **CUR-CO-PP 2** counteract enzymatic activity via precise molecular engagement within the catalytic site, highlighting how strategic linker modification can pivot the mechanism from general antioxidant interference to targeted enzyme inhibition.

Finally, these findings demonstrate that the imaging and anti-melanogenic properties of the curcumin scaffold can be independently optimized through rational molecular design, yielding two distinct functional profiles tailored for different biomedical applications. On one hand, **CUR-C3-PP 1** exhibits selective accumulation, intense fluorescence, and preferential cytotoxicity toward B16-F10 melanoma cells. Despite its moderate tyrosinase inhibition and in vivo depigmenting effects, this specific cellular targeting supports its potential development as a fluorescent tracer for imaging tyrosinase-expressing melanoma. On the other hand, **CUR-CO-PP 2** emerges as a highly promising depigmenting agent; it displays superior anti-melanogenic potency with respect to CUR and KA, and induces robust, dose-dependent in vivo depigmentation in zebrafish embryos with negligible fluorescence. Crucially, cytotoxicity assays and embryonic survival rates confirm that the enhanced efficacy of **CUR-CO-PP 2** does not entail a corresponding increase in cellular or systemic toxicity, validating a mechanism primarily driven by targeted tyrosinase inhibition rather than generalized cellular damage. Future research will focus on the structural optimization of these CUR-based scaffolds to further enhance synthesis yields, alongside preclinical investigations to validate their efficacy in target-specific melanoma imaging and topical depigmenting therapies.

## Figures and Tables

**Table 1:** Inhibitory activity toward *Ab*TYR of piperazinyl phenol-curcumin derivatives **1**–**2** compared to CUR and KA.

Compound	IC_50_ ^a^ Monophenolase (μM ± SD)	IC_50_ ^a^ Diphenolase (μM ± SD)
**CUR-C3-PP 1**	55.34 ± 2.47	51.20 ± 3.69
**CUR-CO-PP 2**	19.92 ± 2.43	12.89 ± 1.66
**CUR**	58.44 ± 1.82	168.57 ± 8.15
**KA**	29.01 ± 0.74	16.03 ± 0.30

^a^ IC_50_ values represent the concentration that caused 50% enzyme activity loss. Compounds were tested in triplicates.

## Data Availability

The original contributions presented in this study are included in the article/Supplementary Material. Further inquiries can be directed to the corresponding authors.

## Supplementary Materials

The following supporting information can be downloaded at: https://www.mdpi.com/article/10.3390/ijms27198759/s1.

## Abbreviations

*Ab*TYR	*Agaricus bisporus* Tyrosinase
ABTS	2,2′-azino-bis(3-ethylbenzothiazoline-6-sulfonic acid)
BODIPY	BOron–DIPYrromethene
CUR	Curcumin
DCM	Dichloromethane
DMF	Dimethylformamide
DMSO	Dimethyl sulfoxide
DPPH	(2,2-diphenyl-1-picrylhydrazyl)
FRAP	Ferric Reducing Antioxidant Power
hpf	Hours Post-Fertilization
*h*TYR	*Human* Tyrosinase
IC_50_	Inhibitory Capacity
KA	Kojic Acid
L-DOPA	L-3,4-dihydroxyphenylalanine
MITF	MIcrophthalmia-associated Transcription Factor
PP	4-(piperazin-1-yl)phenol
PTU	1-phenyl-2-thiourea
TLC	Thin-Layer Chromatography
TRP-1	Tyrosinase-Related Protein 1
TRP-2	Tyrosinase-Related Protein 2
TYR	Tyrosinase
