# Supplementary material for: Curcumin-Based Tyrosinase-Targeting Derivatives for Cell Imaging and Depigmenting Effect
Source: Int J Mol Sci. 2026 Sep 30;27(19):8759. doi: 10.3390/ijms27198759 (PMC13665293; doi:10.3390/ijms27198759)
Supplement: Supplementary file 1 [file ijms-27-08759-s001.zip › ijms-4496784-supplementary.pdf]

Curcumin-Based Tyrosinase-Targeting Derivatives for Cell Imaging and Depigmenting Effect

M. Monforte,[a†] M. Pontoriero,[b†] P.M. Bonaccorsi,[a] A. Calderaro,[a] L. Centorrino,[a,c] V. D'Angelo,[a] L. De Luca,[a] M.P. Germanò,[a] R. Gitto,[a] S. Mirabile,[a*] G.T. Patanè,[a] S. Putaggio,[a] D. Scumaci,[b] C.M.A. Gangemi[a*]

[a] Department of Chemical, Biological, Pharmaceutical, and Environmental Sciences, University of Messina, Viale F. Stagno d'Alcontres 31, 98166, Messina, Italy

[b] Proteomics and Tumor Biochemistry Group, Dpt. of Experimental and Clinical Medicine; School of Medicine, Magna Græcia University of Catanzaro

[c] Fondazione "Prof. Antonio Imbesi", I-98123 Messina, Italy

Table of contents

Chemicals. **2**

Instruments. **2**

Synthesis ***2***

Synthesis of curcumin derivatives 3 and 4 .... **3**

Synthesis of compound 5 .... **3**

Synthesis of hemicurcumin 6 .... **4**

Synthesis of curcumin derivative 4 by modified Pabon procedure **4**

CUR derivative CUR-C3-PP 1 .... **5**

Synthesis of compound 7 .... **5**

Synthesis of compound 8 .... **6**

Synthesis of curcumin derivative CUR-CO-PP 2 .... **6**

In silico studies ***7***

Docking of compounds CUR-C3-PP 1 and CUR-CO-PP 2 as diketo form **7**

Docking of compounds CUR-C3-PP 1 and CUR-CO-PP 2 in enolate form **8**

Docking of compound PP **9**

Docking of Curcumin in diketo, keto-enol and enolate form **10**

^{1}H-NMR and ^{13}C-NMR ***11***

Photophysical characterization of CUR-C3-PP 1 and CUR-CO-PP 2 vs CUR ***16***

Fluorescence imaging studies and cytotoxicity studies ***17***

References ***18***

Chemicals. All Chemicals and solvents were purchased from Merck and, when necessary, purified according to standard procedures. Curcumin was purchased from Merck with purity ≥65% and was further purified by column chromatography using silica gel as stationary phase and $CHCl_3$/*n*-hexane 90/10. All the syntheses were monitored by TLC on commercially available precoated plates (silica gel 60 F_{254}), and the products were visualized with vanillin [1 g dissolved in MeOH (60 mL) and conc. H_2SO_4 (0.6 mL)] under UV lamp illumination. Silica gel 60 was used for column chromatography. Tyrosinase lyophilized powder, ≥1000 unit/mg solid was purchased from Merck and used without purification.

Instruments. Proton (^{1}H) and carbon (^{13}C) NMR spectra were recorded on a Varian 500 spectrometer at 500 MHz for ^{1}H and 125 MHz for ^{13}C using $CDCl_3$, DMSO-d_6. Chemical shifts are given in parts per million (ppm) (δ) relative to $CDCl_3$ δ 7.26 ppm for proton and 77.16 ppm for carbon, relative to DMSO-d_6 δ 2.5 ppm for proton and 39.52 ppm for carbon. Coupling constants (J) are given in hertz, and the attributions are supported by heteronuclear single-quantum coherence (HSQC) and correlation spectroscopy (COSY) experiments. For clarity, the assignment of the ^{1}H NMR signals should be interpreted with reference to the corresponding molecular structures reported in the Supporting Information. Elemental analysis was performed with Eurovector EA3100 CHNS Elemental Analyzers. Melting points (mp) were determined with Buchi B-545 apparatus (BUCHI Labortechnik AG Flawil, Switzerland) and are uncorrected. A stereomicroscope (SMZ-171 Series, Motic) equipped with a digital camera (Moticam® 5 plus) was used for image acquisition. Luminescence was recorded using a GloMax luminometer (Promega, Madison, WI, USA). Leica Stellaris 5 confocal microscope (Leica Microsystems, Wetzlar, Germany) equipped with a 63× oil immersion objective.

Synthesis

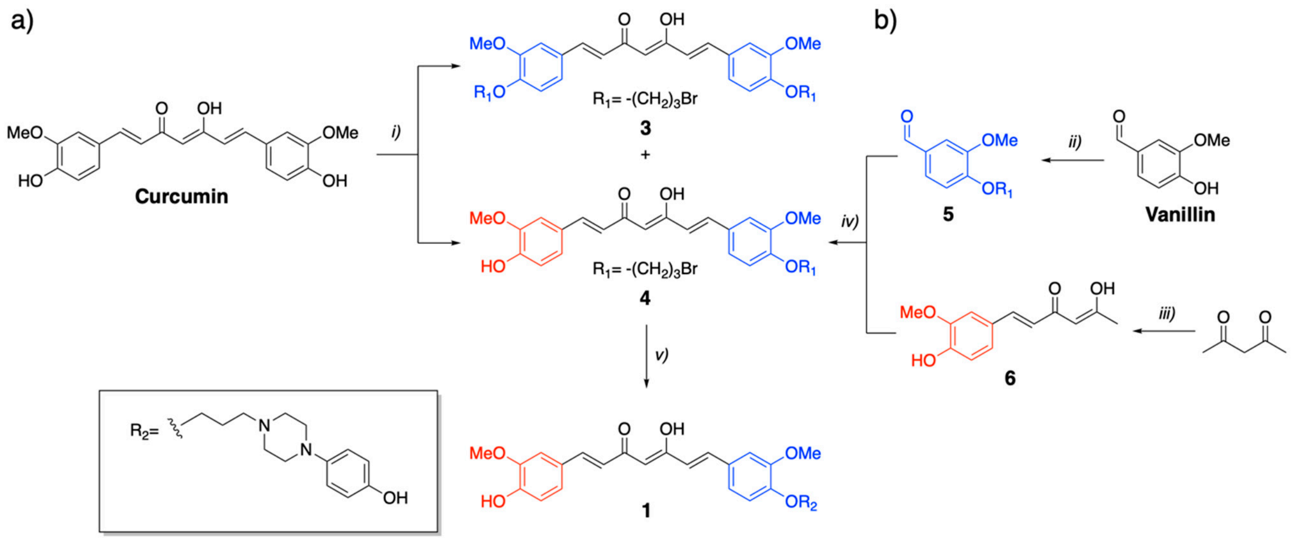

Scheme S1. Reagents and conditions: *i)* $Br(CH_2)_3Br$, K_2CO_3, dry acetone, rt; *ii)* $Br(CH_2)_3Br$, K_2CO_3, dry DMF, 80°C ; *iii)* B_2O_3, ethyl acetate, 80°C, 30 min; then vanillin and (*n*-BuO)$_3$B in ethyl acetate, 30 min, then *n*-BuNH$_2$, 30 min; then 1 N HCl, 50 °C, 30 min. *iv)* B_2O_3, ethyl acetate, 80°C, 30 min, then compound **5** and (*n*-BuO)$_3$B in ethyl acetate, 30 min, then piperidine, 30 min, then 0.4 N HCl, 50 °C, 30 min; *v)* 4-(piperazin-1-yl)phenol (**PP**), $NaHCO_3$, dry acetonitrile, reflux.

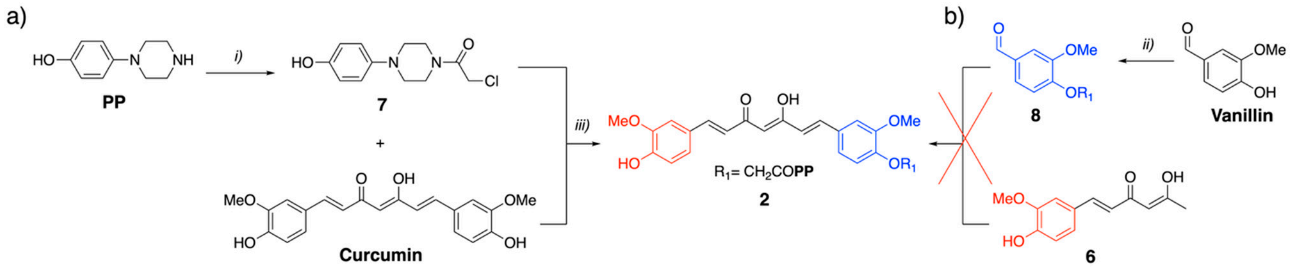

Scheme S2. Reagents and conditions: *i)* 2-Chloroacetyl chloride, dry DMF, 0°C to rt; *ii)* compound **7**, K_2CO_3, dry DMF, 80°C; *iii)* K_2CO_3, dry DMF, 80°C;

Synthesis of curcumin derivatives 3 and 4

Curcumin (200 mg, 0.540 mmol), K_2CO_3 (74.4 mg, 0.540 mmol) and 1,3-dibromopropane (46 µL, 0.540 mmol) were dissolved in 10 mL of dry acetone. The reaction mixture was stirred at room temperature and under inert atmosphere overnight. The solvent was removed under reduced pressure, and the crude was dissolved in ethyl acetate and washed with water (3 x 50 mL). The organic phases were collected, dried over anhydrous Na_2SO_4 and filtered. The solvent was removed under reduced pressure and the crude was purified by column chromatography (SiO_2; $CHCl_3$ 100% to $CHCl_3$/methanol 98:2) obtaining compound **3** [18] (*n*-hexane/Ethyl Acetate 6:3 R_f=0.38; 33 mg, 10%) and compound **4** [19] (*n*-hexane/Ethyl Acetate 6:3 R_f=0.21; 50 mg, 19%) both as yellow powder.

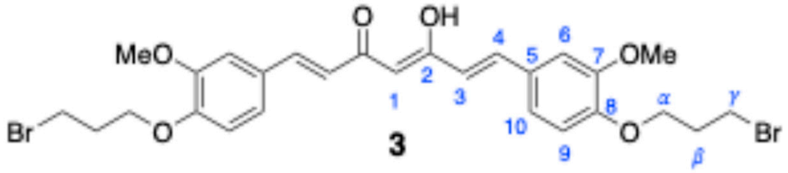

Compound **3** ^{1}H NMR (500 MHz, $CDCl_3$) δ:7.60 (d, *J*= 15.5 Hz, 2H, H-4; 7.15-7.11 (m, 2H, H-10); 7.08 (d, *J*= 2.0 Hz, 2H, H-6); 6.92 (d, *J*= 8.2 Hz, 2H, H-9); 6.50 (d, *J*= 15.5 Hz, 2H, H-3); 5.82 (s, 1H, H-1); 4.20 (t, *J*= 6.0 Hz, 4H, H-α); 3.91 (s, 6H, -OMe); 3.63 (t, *J*= 6.0 Hz, 4H, H-γ); 2.39 (p, *J*= 6.0 Hz, 4H, H-β). Characterization data are limited to ^{1}H-NMR analysis as the compound is known and was not used in onward synthesis.

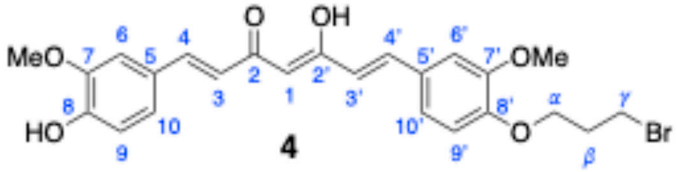

Compound **4** ^{1}H NMR (500 MHz, $CDCl_3$) δ: 7.60 and 7.59 (2x d, *J*= 16 Hz, 2H, H-4 and H-4'); 7.15-7.10 (m, 2H, H-10 and H-10'); 7.08 and 7.05 (2x d, *J*= 2.0 Hz, 2H, H-6 and H-6'); 6.93 and 6.91 (2x d, *J*= 8.2 Hz, 2H, H-9 and H-9'); 6.49 and 6.48 (2x d, *J*= 16 Hz, 2H, H-3 and H-3'); 5.81 (s, 2H, H-1 and OH); 4.19 (t, *J*= 6.0 Hz, 2H, H-α); 3.94 and 3.90 (2x s, 6H, -OMe); 3.63 (t, *J*= 6.4 Hz, 2H, H-γ); 2.39 (p, *J*= 6.1Hz, 2H, H-β). Characterization data are limited to ^{1}H-NMR analysis as the compound is known

Synthesis of compound 5

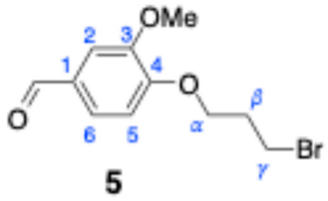

Vanillin (1 g, 6.6 mmol) and K_2CO_3 (1.27 g, 9.2 mmol) were added to 1,3-dibromopropane (19.7 mmol) in dry DMF (50 mL). The reaction mixture was stirred at 80 °C for 1 h under inert atmosphere and monitored by TLC until the disappearance of the starting compound (R_f =0.61 *n*-Hexane: Ethyl acetate 6:4). The reaction mixture was cooled on an ice bath and quenched with 1 N HCl. After extraction with ethyl acetate (3x 50 mL), the organic layers were collected, washed with water, dried over anhydrous Na_2SO_4 and filtered. The solvent was removed under reduce pressure, and the crude was purified by column chromatography (SiO_2; *n*-hexane−ethyl acetate 7/2 to 6/3) affording compound **5** [19] (R_f =0.83 *n*-Hexane: Ethyl acetate 6:4; mp 58.3−59.8 °C; 1.1 g, 61%) as off-white solid. ^{1}H NMR (500 MHz, $CDCl_3$) δ: 9.85 (s, 1H, -CHO), 7.46-7.42 (dd, *J*= 8.2 Hz, *J*= 1.8 Hz, 1H, H-6); 7.41 (d, *J*= 1.8 Hz, 1H, H-2); 7.00 (d, *J*= 8.2 Hz,1H, H-5); 4.24 (t, *J*= 6.0 Hz, 2H, H-α); 3.91(s, 3H, -OMe) 3.64 (t, *J*= 6.3 Hz, 2H, H-γ), 2.45- 2.35 (m, 2H, H-β). Characterization data are limited to ^{1}H-NMR analysis as the compound is known.

Synthesis of hemicurcumin 6

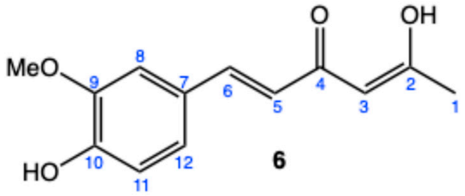

6

2,4-Pentanedione (1.62 mL, 15.8 mmol) and B_2O_3 (1.0 g, 14.4 mmol) were dissolved in ethyl acetate (10 mL) and the solution was stirred at 80 °C for 30 min. A solution of vanillin (1.1 g, 7.19 mmol) and (*n*-BuO)$_3$B (0.8 mL, 2.9 mmol) in 10 mL of ethyl acetate was added to the reaction mixture. After stirring for 30 min at 80 °C, *n*-butylamine (0.28 mL, 2.9 mmol) was added dropwise and allowed to stir at 80 °C for 1 h. An aqueous solution of HCl (1N, 10 mL) was added to the mixture and stirred at 50 °C for 30 min. The reaction mixture was extracted with ethyl acetate, washed with water, and then dried over anhydrous Na_2SO_4, and filtered. The solvent was removed under reduced pressure, and the crude was purified by column chromatography (SiO_2; *n*-hexane/ethyl acetate 2:1). The fractions, containing the desired compound, were collected and crystallized from ethanol and water, giving pure compound **6** [19] (706 mg; 42%) as a yellow crystallin powder (R_f 0.53 *n*-Hexane/Ethyl acetate 2/1); mp 143.7−145.9 °C; ^{1}H NMR (500 MHz, $CDCl_3$) δ: 7.53 and 6.32 (2x d, *J*= 15.8 Hz, 2x 1H, H-5 and H-6); 7.09 (dd, *J*= 8.2 Hz, *J*= 1.9 Hz, 1H, H-12); 7.02 (d, *J*= 1.9 Hz, 1H, H-8); 6.92 (d, *J*= 8.2 Hz, 1H, H-11); 5.84 and 5.63 (2x s, 1H, H-3 and OH); 3.94 (s, 3H, OCH_3); 2.16 (s, 3H, H-1). Characterization data are limited to ^{1}H-NMR analysis as the compound is known.

Synthesis of curcumin derivative 4 by modified Pabon procedure

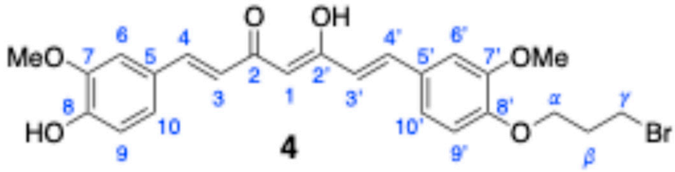

4

Compound **5** (260.0 mg, 1.11 mmol) and B_2O_3(114.2 mg, 1.64 mmol) were dissolved in ethyl acetate (5 mL) and stirred at 80 °C for 30 min. To this mixture an ethyl acetate solution (5 mL) of **6** (178 mg, 0.65 mmol) and (*n*-BuO)$_3$B (0.44 mL, 1,6 mmol) was added and the reaction mixture was stirred at 80°C for 30 min. Then, piperidine (39 μL, 0.39 mmol) was added and the reaction was stirred at 80 °C for 30 min. An aqueous solution HCl (0.4 M, 5 mL) was added to the mixture that stirred at 50 °C for 30 min. Extraction with ethyl acetate, washing with water and drying over

anhydrous Na_2SO_4 gave the crude that was purified by column chromatography (SiO_2; n-hexane/ethyl acetate; 2:1) to afford compound **4** [19] (112 mg, 37%) as a yellow solid. Characterization of compound **4** has been previously reported in this Supplementary Materials.

CUR derivative CUR-C3-PP 1

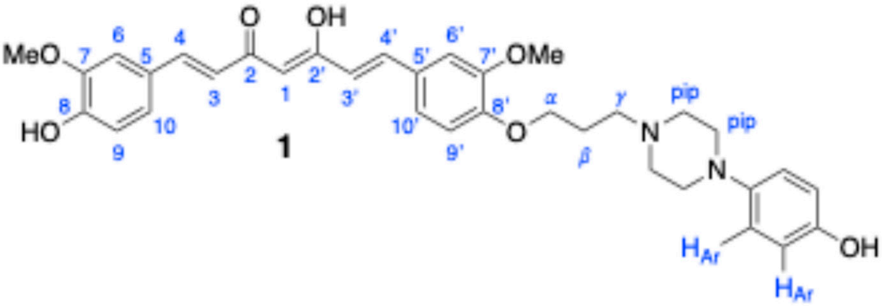

Yellow solid. Rf= 0.33 eluant $CHCl_3$/MeOH 9:1;mp= 122-124°C. ^{1}H NMR (500 MHz, DMSO-d_6) δ: 7.56 and 7.55 (2x d, *J*= 15.5 Hz, 2x 1H, H-4 and H-4'); 7.34 and 7.32 (2x d, *J*= 2.0 Hz, 2x 1H, H-6 and H-6'); 7.24 and 7.15 (2x dd; *J*= 8.5 Hz, *J*= 2.0 Hz, 2x 1H, H-10 and H-10'); 7.02 (d, *J*= 8.5 Hz, 1H, H-9'); 6.86-6.71 (m, 3H, H-9, H-3, H-3'); 6.77 and 6.64 (AA'BB' system, *J*= 8.9 Hz, 4H, H_{ar}); 6.08 (s, 1H, H-1); 4.07 (t, *J*=6.4 Hz, 2H, H-α); 3.84 (s, 6H, 2x -OMe); 3.02-2.88 (m, 4H, H_{pip}); 2.57-2.37 (m, 6H, H_{pip} and H-γ);1.91 (m, 2H, H-β).^{13}C NMR (126 MHz, DMSO-d_6) δ:184.1; 183.1; 151.3; 150.7; 150.0 149.6; 148.5; 144.7; 141.4; 140.6; 128.0; 126.6; 123.7; 123.3; 122.5; 121.5; 118.1; 116.2; 115.9; 113.2; 111.8; 111.1; 101.4; 67.1; 56.11; 54.8; 55.3; 50.4; 26.2. Anal. For ($C_{34}H_{38}N_2O_7$) C 69.61, H 6.53, N 4.77, O, 19.09, Found: C 69.58; H 6.56, N 4.73.

Synthesis of compound 7

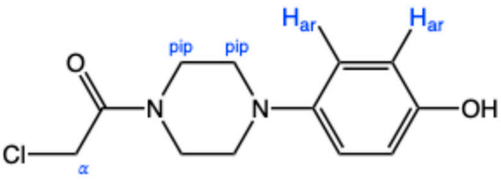

7

2-Chloroacetyl chloride (90 µL, 1.12 mmol) was added slowly at 0°C to a solution of 4-(piperazin-1-yl)phenol (**PP**) (200 mg, 1.12 mmol) in dry DMF (4 mL). The reaction mixture was stirred, under inert atmosphere, and at room temperature for 1 hr, until the disappearance of reactant. A saturated solution of $NaHCO_3$ (5 mL) was added to quench the reaction mixture that was extracted with ethyl acetate (3x 15mL). The organic layers were collected, dried over anhydrous Na_2SO_4, filtered and the solvent was removed under vacuum. The desired compound **7** [21] was crystallized from Et_2O/ EtOH. (R_f =0.40 eluant CH_2Cl_2:MeOH 96:4; m.p.: 162–163 °C; 242 mg, 67%).^{1}H NMR (500MHz, DMSO-d_6) δ: 8.88 (bs, 1H, OH); 6.81 and 6.64 (AA'BB' system, *J*= 8.2Hz, 4H, H_{Ar}); 4.40 (s, 2H, H-α), 2.40–2.50 and 3.30-3.42 (2x m, 2x 4H, H_{pip}). Characterization data are limited to ^{1}H-NMR analysis as the compound is known.

Synthesis of compound 8

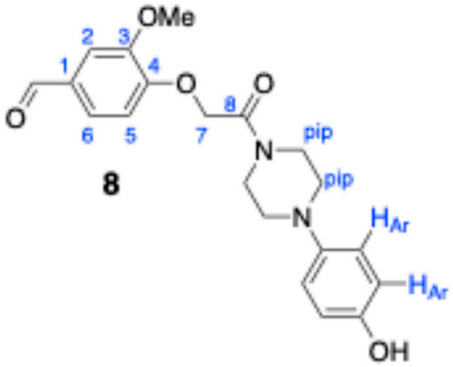

Vanillin (107 mg, 0.70 mmol), compound **7** (150 mg, 0.58 mmol) and K_2CO_3 (80 mg, 1.16 mmol) were dissolved in dry DMF (15 mL), and the mixture was stirred, under inert atmosphere at 80°C overnight. The solvent was removed under reduced pressure, and the crude was dissolved in ethyl acetate and washed with water (3 x 50 mL). The organic layers were collected, dried over anhydrous Na_2SO_4, filtered and the solvent was removed under vacuum. The crude was purified by column chromatography (SiO_2, *n*-Hexane/Ethyl acetate 2:8) affording the desired compound **8** (R_f =0.35 ethyl acetate/*n*-Hexane 8/2; 75 mg, 35 %). ^{1}H NMR (500 MHz, $CDCl_3$) δ: 9.86 (s, 1H, CHO); 7.45-7.41 (m, 2H, H-2 and H-5); 7.07 (dd, *J*=1.0 Hz, *J*= 7.5 Hz, H-6); 6.82 and 6.77 (AA'BB' system, 4H, H_{Ar}); 4.89 (s, 2H, H-7); 3.93 (s, 3H, -OMe); 3.83-3.69 (m, 4H, H_{pip}) and 3.10-2.92 (m, 4H, H_{pip}). ^{13}C NMR (125 MHz, $CDCl_3$) δ: 191.0; 165.8, 150.5, 150.1, 141.3, 131.2, 119.4, 116.1, 112.8, 112.7, 68.5, 56.3, 56.1, 51.6, 51.0, 45.6.

Synthesis of curcumin derivative CUR-CO-PP 2

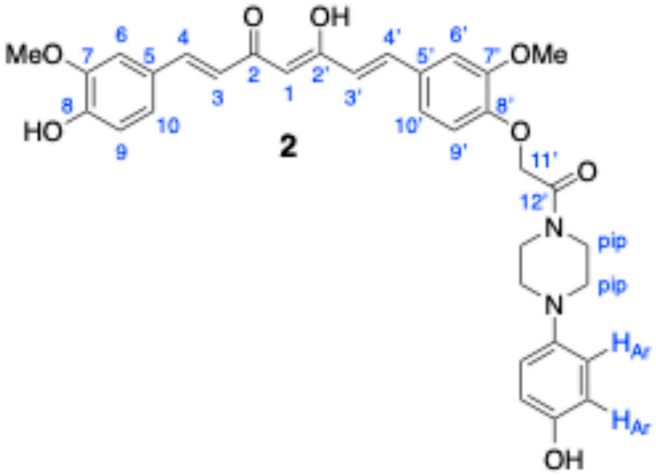

Yellow solid, R_f =0.30, CH_2Cl_2/MeOH 96:4; mp 114-116 °C. ^{1}H NMR (500 MHz, $CDCl_3$) δ: 7.59 and 7.58 (2x d, *J*= 15.5 Hz, 2H, H-3 and H-3'); 7.15-7.09 (m, 2H, H-10 and H-10'); 7.08 and 7.05 (2x d, *J*= 2.0 Hz, 2H, H-6 and H-6'); 6.97 and 6.93 (2x d, *J*= 8.0 Hz, 2H, H-9 and H-9'); 6.83 and 6.78 (AA'BB' system, 4H, H_{ar}); 6.49 and 6.48 (2x d, *J*= 15.5 Hz, 2H, H-4 and H-4'); 5.88 and 5.82 (2x s, 2H, H-1 and OH); 4.83 (s, 2H, H-11); 3.95 and 3.92 (2x s, 6H, -OMe); 3.83-3.70 (m, 4H H_{pip}) and 3,09-2.97 (m, 4H, H_{pip}). ^{13}C NMR (126 MHz, $CDCl_3$) δ: 183.9, 182.9, 166.3, 150.5, 149.8, 149.8, 148.1, 147.0, 145.4, 140.9, 140.1, 129.6, 127.8, 123.1, 122.8, 122.3, 121.9, 119.3, 116.1, 115.0, 113.9, 110.8, 109.8, 110.2, 101.4, 68.8, 56.1, 52.6, 52.0, 45.6, 42.5. Anal. For ($C_{33}H_{34}N_2O_8$) C 67.56, H 5.84, N 4.78, O 21.82. Found: C 67.53, H 5.76, N 4.81.

In silico studies

Docking of compounds CUR-C3-PP 1 and CUR-CO-PP 2 as diketo form

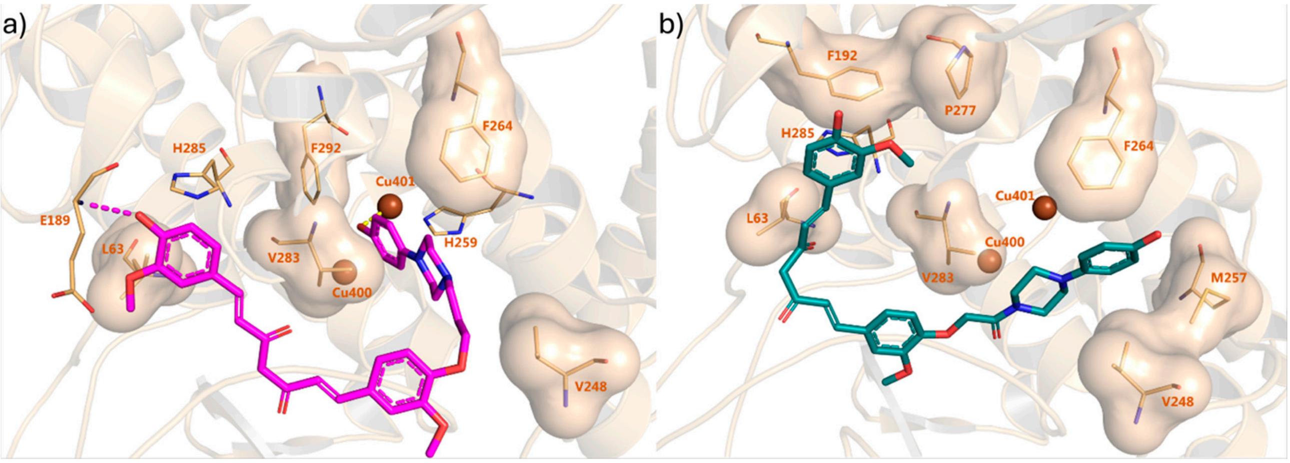

Figure S1. Three-dimensional representation of the molecular docking studies of **CUR-C3-PP 1**(panel a) and **CUR-CO-PP 2**(panel b) in diketo form within the *Ab*TYR active site (RCSB PDB: 2Y9X). [27] The protein backbone is depicted as a wheat cartoon structure, while copper ions are represented as brown spheres. Aminoacidic residues involved in hydrophobic interaction are displayed as surface representations, while those participating in other protein–ligand interactions are shown as sticks. The ligands as represented as sticks coloured in magenta (**CUR-C3-PP 1,** a) and deep teal (**CUR-CO-PP 2**, b). Coordination bonding interactions are depicted as yellow dashed lines. The image was generated using PyMOL.

CUR-C3-PP 1 in its diketo form can fit into the catalytic cavity containing the Cu ions through its pharmacophoric moiety (**PP**), establishing a coordination bond with Cu401. The aromatic ring of **PP** forms π–π interactions with H259, while the aromatic ring of the curcumin scaffold establishes π–π interactions with H285. In addition, the molecule forms a hydrogen bond between the phenolic hydroxyl group of the curcumin moiety and the nitrogen atom of E189. Furthermore, **CUR-C3-PP 1** establishes hydrophobic interactions with L63, V248, F259, V283, and F292.

CUR-CO-PP 2 in its diketo form is unable to penetrate the catalytic cavity containing the Cu ions; however, it is still capable of establishing several interactions with the enzyme. The aromatic ring of the curcumin moiety forms π–π interactions with H285. In addition, the molecule establishes hydrophobic interactions with several amino acid residues, including L63, F192, V248, M257, F264, P277 and V283.

The docking score of the best-ranking poses were 47.57 for **CUR-C3-PP 1** and 39.16 for **CUR-CO-PP 2.**

Binding free energy (ΔG_binding) calculations were performed for the ligand–protein complexes, yielding a value of −46.59 and -43.55 for **CUR-C3-PP 1** and **CUR-CO-PP 2**, respectively.

Docking of compounds CUR-C3-PP 1 and CUR-CO-PP 2 in enolate form

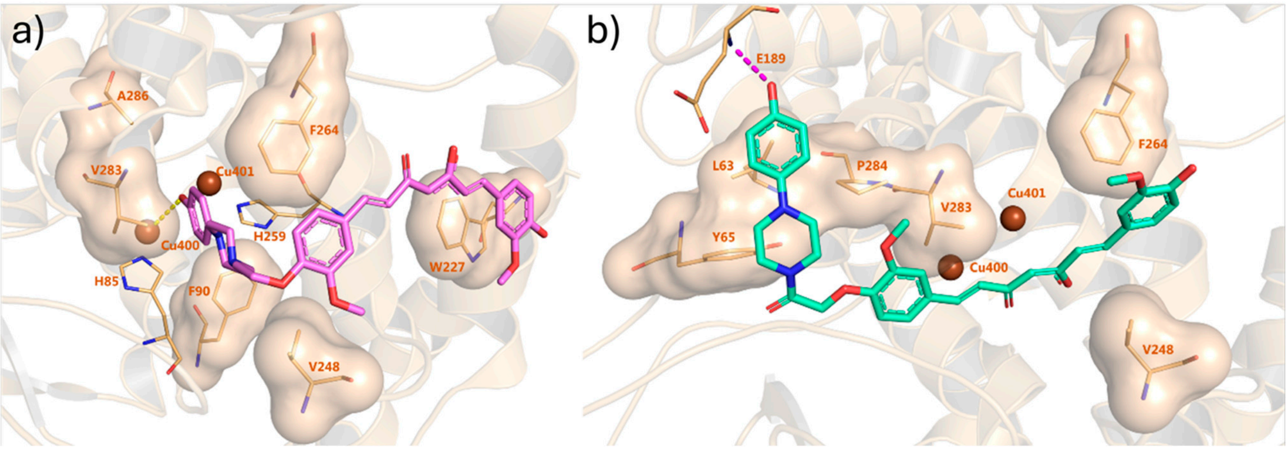

Figure S2. Three-dimensional representation of the molecular docking studies of **CUR-C3-PP 1**(panel a) and **CUR-CO-PP 2** (panel b) in enolate form within the *Ab*TYR active site (RCSB PDB: 2Y9X). [27] The protein backbone is depicted as a wheat cartoon structure, while copper ions are represented as brown spheres. Aminoacidic residues involved in hydrophobic interaction are displayed as surface representations, while those participating in other protein–ligand interactions are shown as sticks. The ligands as represented as sticks coloured in magenta (**CUR-C3-PP 1,** a) and deep teal (**CUR-CO-PP 2**, b). Coordination bonding interactions are depicted as yellow dashed lines. The image was generated using PyMOL.

The docking analysis provides a structural rationale for the distinct inhibitory profiles exhibited by the two anionic derivatives. The enolate form of **CUR-C3-PP 1** (figure S2a) adopts a binding orientation that positions the **PP** within the catalytic pocket of *Ab*TYR, allowing the phenolic hydroxyl group to establish a direct interaction with the Cu400 ion. This positioning is particularly relevant because the copper centers constitute a key structural and functional element of the tyrosinase catalytic machinery. The proximity of the phenolic group to Cu400 may therefore allow the enolate form of **CUR-C3-PP 1** to directly perturb the local coordination environment of the catalytic metal and potentially interfere with substrate positioning and/or the catalytic cycle. The binding mode is further stabilized by π–π interactions involving the aromatic ring and H85 and H259, residues located in the vicinity of the catalytic pocket, together with a broad hydrophobic interaction network involving F90, W227, V248, F264, V283, and A286. These additional contacts may contribute to anchoring the ligand in a defined orientation, thereby favouring persistence of the phenolic moiety close to the copper center. Thus, the docking pose suggests that the enolate form of **CUR-C3-PP 1** may inhibit *Ab*TYR through a combination of direct catalytic-metal engagement and complementary aromatic/hydrophobic interactions, providing a structural basis for interference with the catalytic site.

In contrast, the enolate form of **CUR-CO-PP 2** (figure S2b) displays a markedly different binding mode, as the ligand does not establish direct contacts with either catalytic copper ion. Its interaction with *Ab*TYR appears to be dominated by non-metal-mediated contacts, including a hydrogen bond with E189 and π–π/hydrophobic interactions involving L63, Y65, V248, F264, V283, and P284. The absence of direct copper coordination is structurally consistent with the experimentally determined non-competitive inhibition profile, since ligand binding does not necessarily require occupation or direct perturbation of the catalytic metal center. The interaction network observed for the enolate form of **CUR-CO-PP 2** may instead stabilize the ligand in an adjacent or alternative region of the binding cavity, potentially inducing local conformational or dynamic changes that reduce catalytic efficiency without directly competing with substrate binding at the copper center. In particular, the hydrogen bond with E189 may provide a directional anchoring interaction, while the aromatic and hydrophobic contacts could reinforce the ligand's residence within the protein and constrain its local orientation.

Collectively, the docking results therefore support a binding-mode-dependent inhibition mechanism, in which the enolate form of **CUR-C3-PP 1** is structurally positioned to directly engage the catalytic machinery of *Ab*TYR, whereas the enolate form of **CUR-CO-PP 2** relies on a distinct interaction network that is compatible with inhibition through an alternative, non-copper-coordinating binding mode. Although docking alone cannot establish the precise inhibitory mechanism, the contrasting poses provide a coherent structural hypothesis linking the observed residue-level interactions to the experimentally determined inhibition behaviour of the two compounds.

The docking scores of the best-ranked poses identified by the program are 51.90 for the enolate form of **CUR-C3-PP 1** and 41.71 for the enolate form of **CUR-CO-PP 2**.

ΔG_binding values of -55.28 and -51.58 were obtained for the enolate form of **CUR-C3-PP 1** and the enolate form of **CUR-CO-PP 2,** respectively.

Docking of compound PP

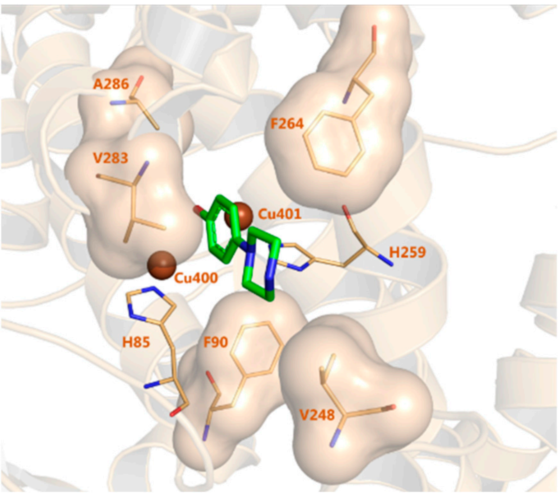

Figure S3. Three-dimensional representation of the molecular docking studies of pharmacophore **PP** within the *Ab*TYR active site (RCSB PDB: 2Y9X).[27] The protein backbone is depicted as a wheat cartoon structure, while copper ions are represented as brown spheres. Amino acidic residues involved in hydrophobic interaction are displayed as surface representations, while those participating in other protein–ligand interactions are shown as sticks. The ligands are represented as sticks coloured in green. The image was generated using PyMOL.

The **PP** pharmacophore is accommodated within the protein cavity, adopting a binding mode that enables favourable π–π interactions with H85 and H259, two histidine residues involved in the coordination of the catalytic Cu ions. These interactions contribute to the positioning and stabilization of the aromatic moiety within the active site. In addition, the pharmacophore establishes an extensive network of hydrophobic contacts with F90, V248, F264, V283, and A286. These residues are located within the catalytic pocket and provide a hydrophobic environment that is likely to contribute to ligand recognition and stabilization of the bound conformation. Overall, the combination of π–π and hydrophobic interactions suggests that the **PP** moiety plays a key role in anchoring the ligand within the catalytic site and may contribute substantially to its inhibitory activity. The docking score of the best-ranked pose identified by the program is 28.58. Binding free energy (ΔG_binding) calculations were performed for the ligand–protein complex, yielding a value of −31.59.

Docking of Curcumin in diketo, keto-enol and enolate form

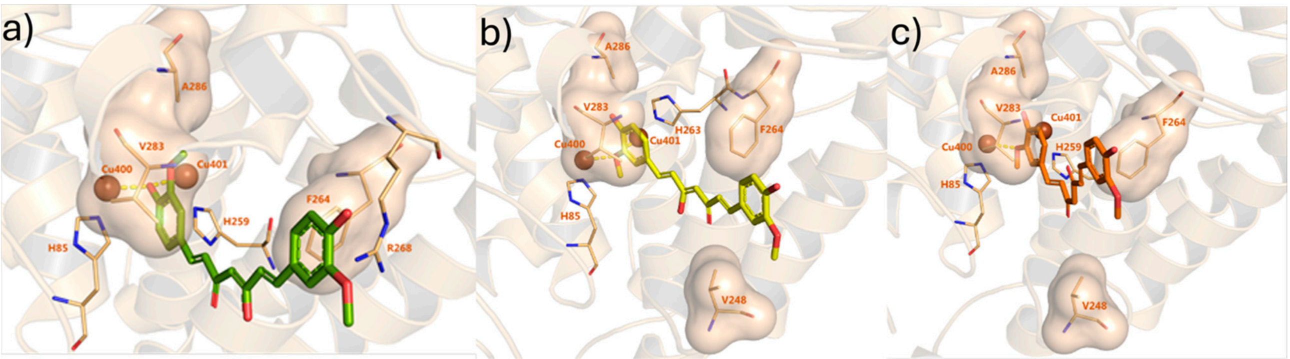

Figure S4. Three-dimensional representation of the molecular docking studies of CUR diketo form (panel a), CUR keto-enol form (panel b) and CUR anionic form (c) within the *Ab*TYR active site (RCSB PDB: 2Y9X).[27] The protein backbone is depicted as a wheat cartoon structure, while copper ions are represented as brown spheres. Amino acidic residues involved in hydrophobic interaction are displayed as surface representations, while those participating in other protein–ligand interactions are shown as sticks. The ligands are represented as sticks coloured in green (CUR diketo form, a), yellow (CUR keto-enol form, b) and orange (CUR anionic form, c). Coordination bonding interactions are depicted as yellow dashed lines. The image was generated using PyMOL.

Molecular docking simulations were performed for both tautomeric forms of CUR, which exhibited highly similar binding modes and interaction profiles within the active site. CUR was found to penetrate deeply into the Cu-containing catalytic site, establishing hydrophobic interactions with residues V248, F264, V283, and A286, as well as π–π stacking interactions with H85. The diketo tautomer additionally formed π–π stacking interactions with H259 and a coordination bond with Cu400. In contrast, the keto-enol tautomer established an additional π–π interaction with H263 and coordination bonds with both copper ions. Overall, both tautomeric forms displayed a comparable binding orientation within the catalytic pocket. The diketo tautomer exhibited a docking score of 34.16, whereas the keto-enol tautomer showed a docking score of 30.14. The binding free energy (ΔG_binding) calculations yielded values of -40.07 and -40.24 for the diketo and keto-enol tautomeric forms of CUR, respectively.
The anionic form of CUR can penetrate the catalytic site of the protein, where the ligand adopts a conformation resembling the diketo and keto–enol tautomers of CUR. The molecule establishes a coordination bond with the Cu ions through its phenolic group. In addition, hydrophobic interactions are established with residues F264, V283, and A286 within the catalytic site. The aromatic ring inserted into the catalytic pocket engages in π–π interactions with residues H85 and H259, whereas the second aromatic ring establishes a π–cation interaction with R268. The docking score of the best-ranked pose identified by the program is 36.27. Binding free energy (ΔG_binding) calculations were performed for the ligand–protein complex, yielding a value of −45.73.

^{1}H-NMR and ^{13}C-NMR

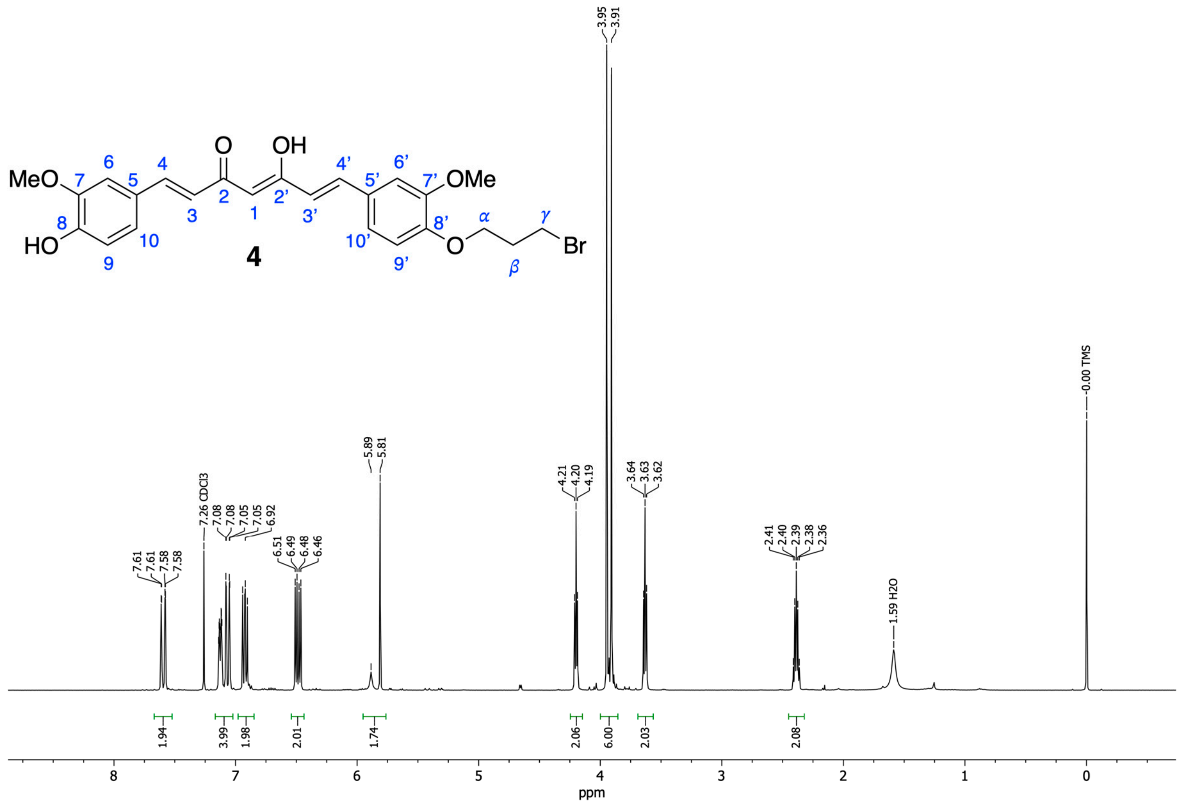

Figure S5. ^{1}H-NMR of compound **4** in $CDCl_3$

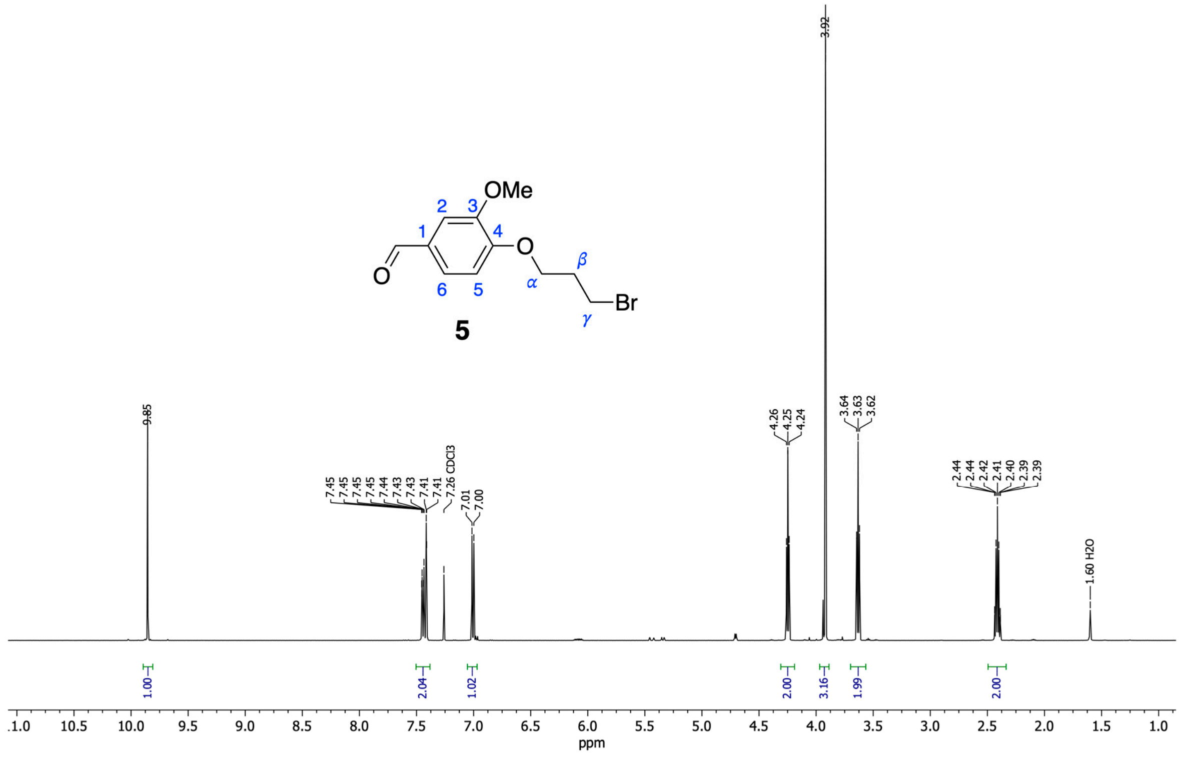

Figure S6. ^{1}H-NMR of compound **5** in $CDCl_3$

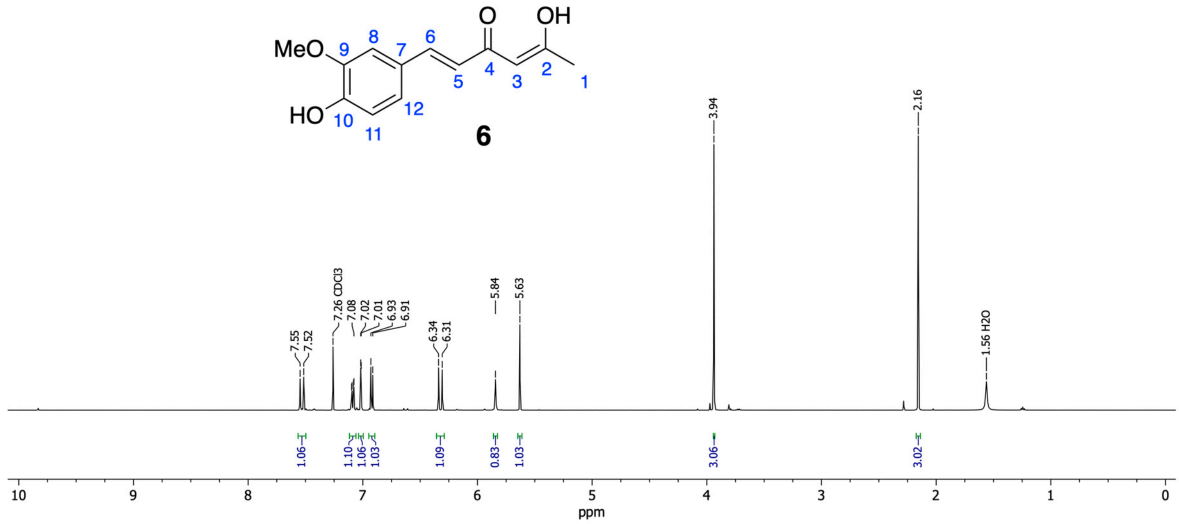

Figure S7. ^{1}H-NMR of compound **6** in $CDCl_3$

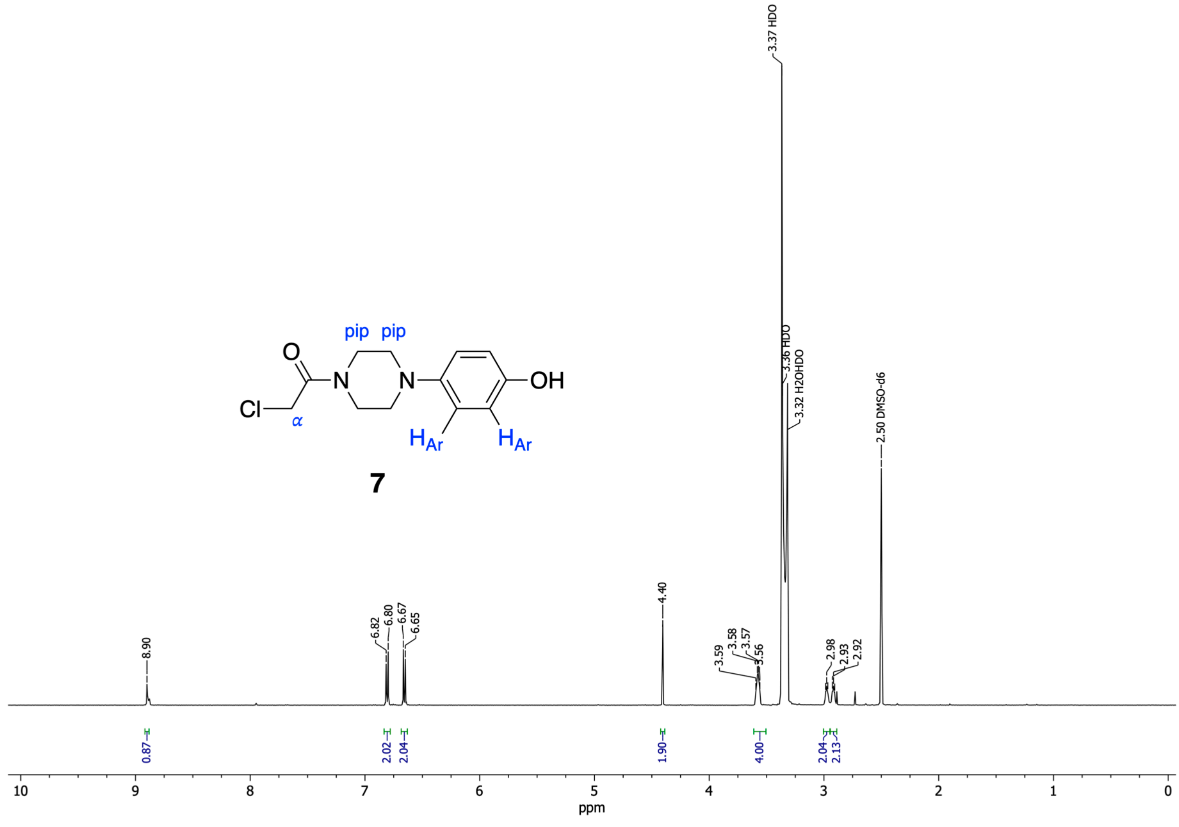

Figure S8. ^{1}H-NMR of compound **7** in DMSO-*d6*

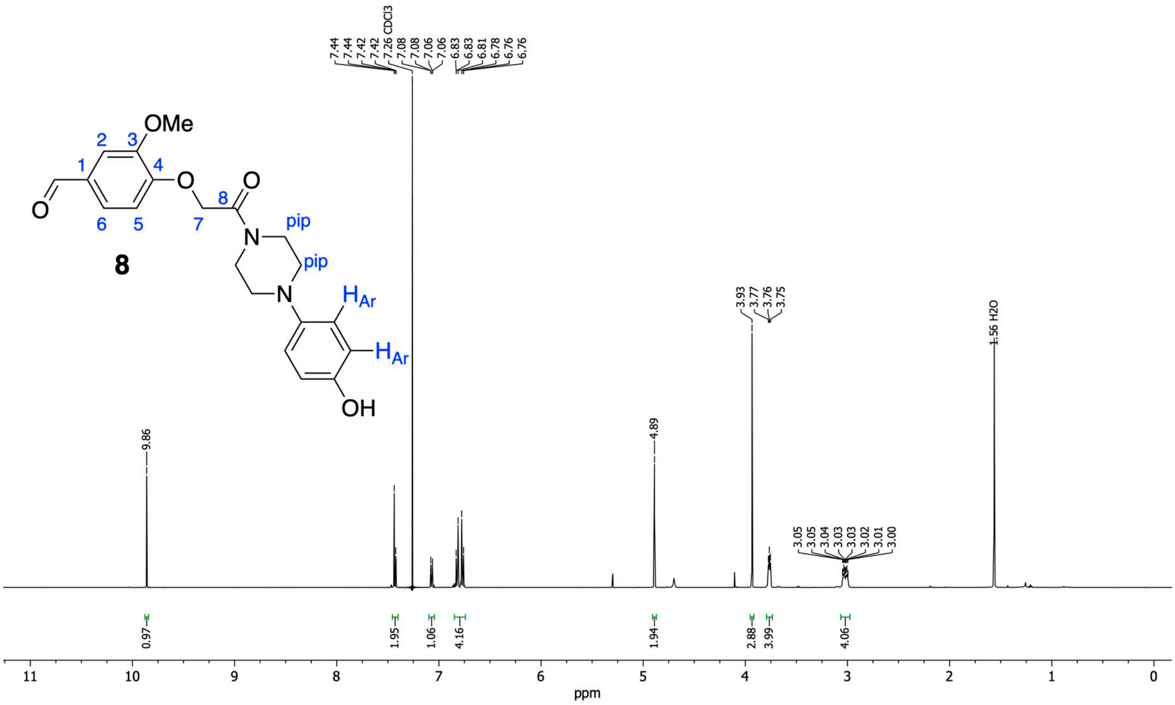

Figure S9. ^{1}H-NMR of compound **8** in $CDCl_3$

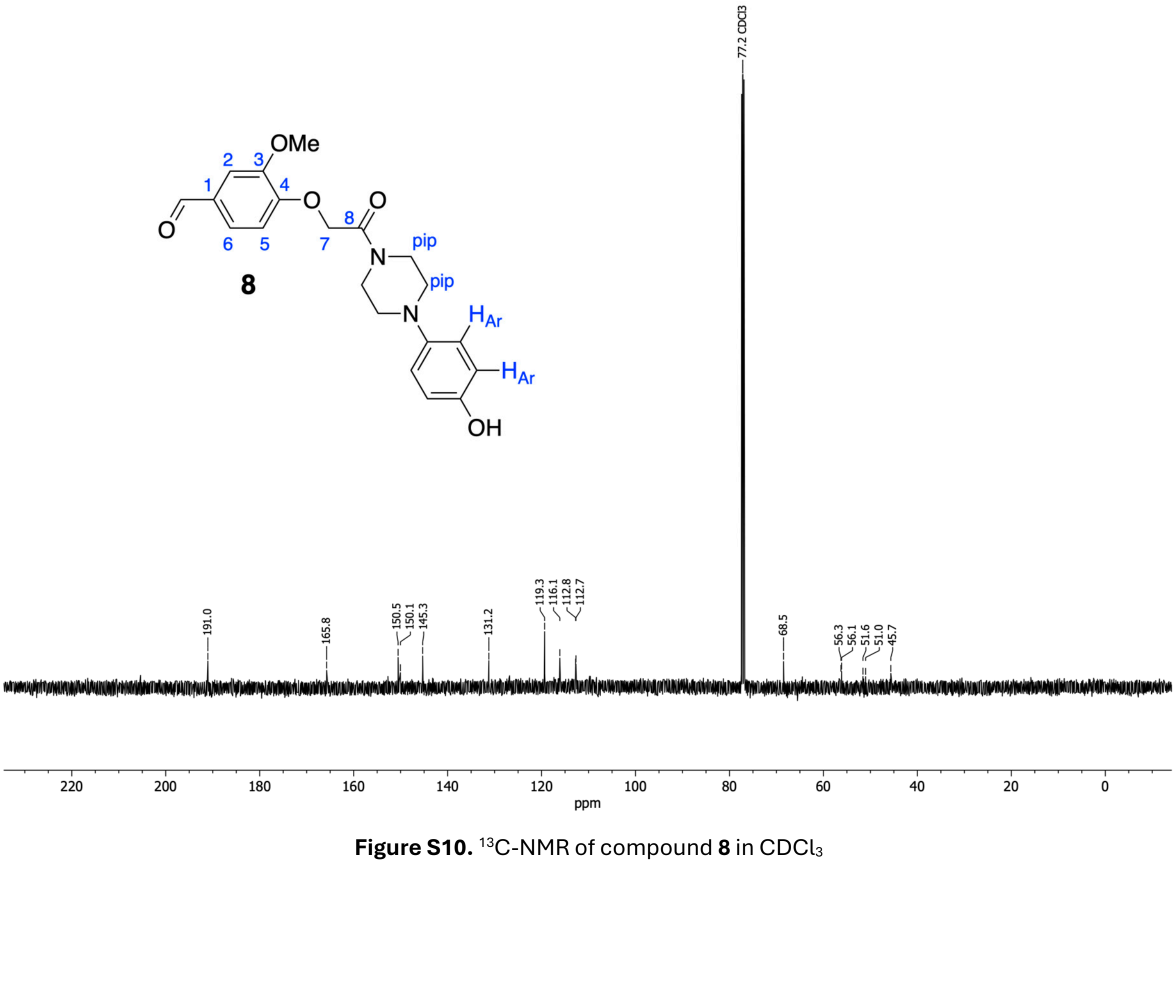

Figure S10. ^{13}C-NMR of compound **8** in $CDCl_3$

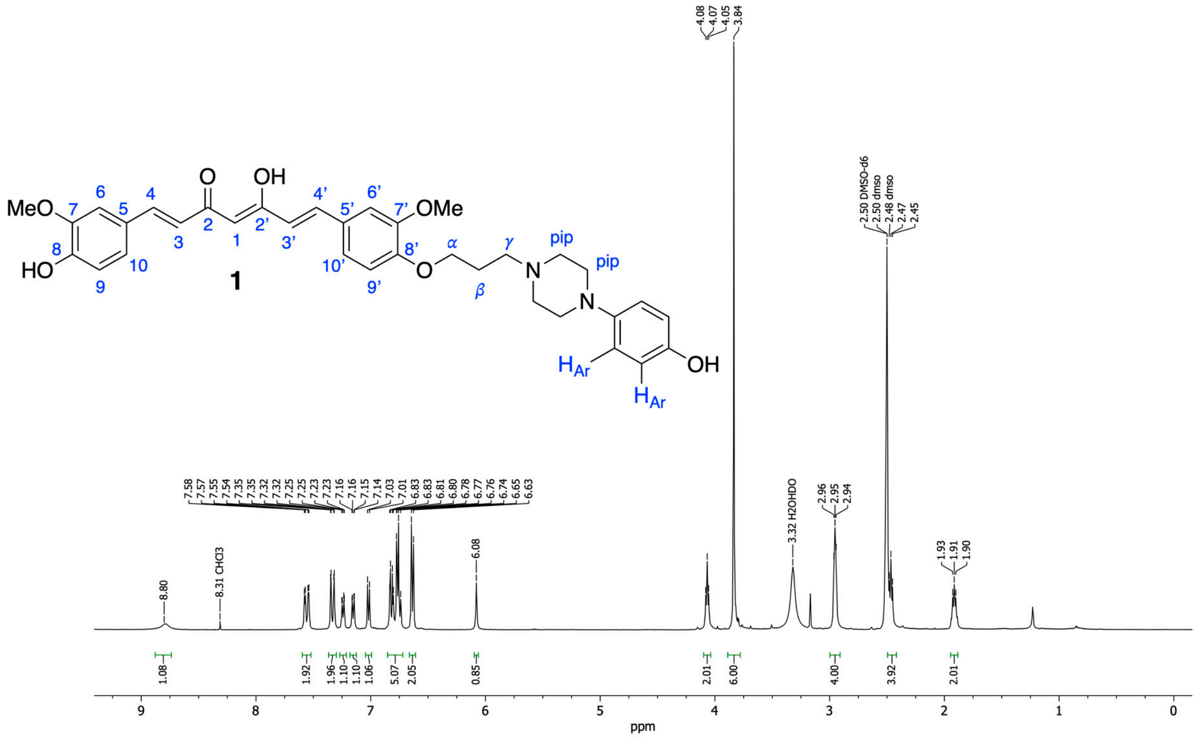

Figure S11. ^{1}H-NMR of compound **1** in DMSO-*d6*

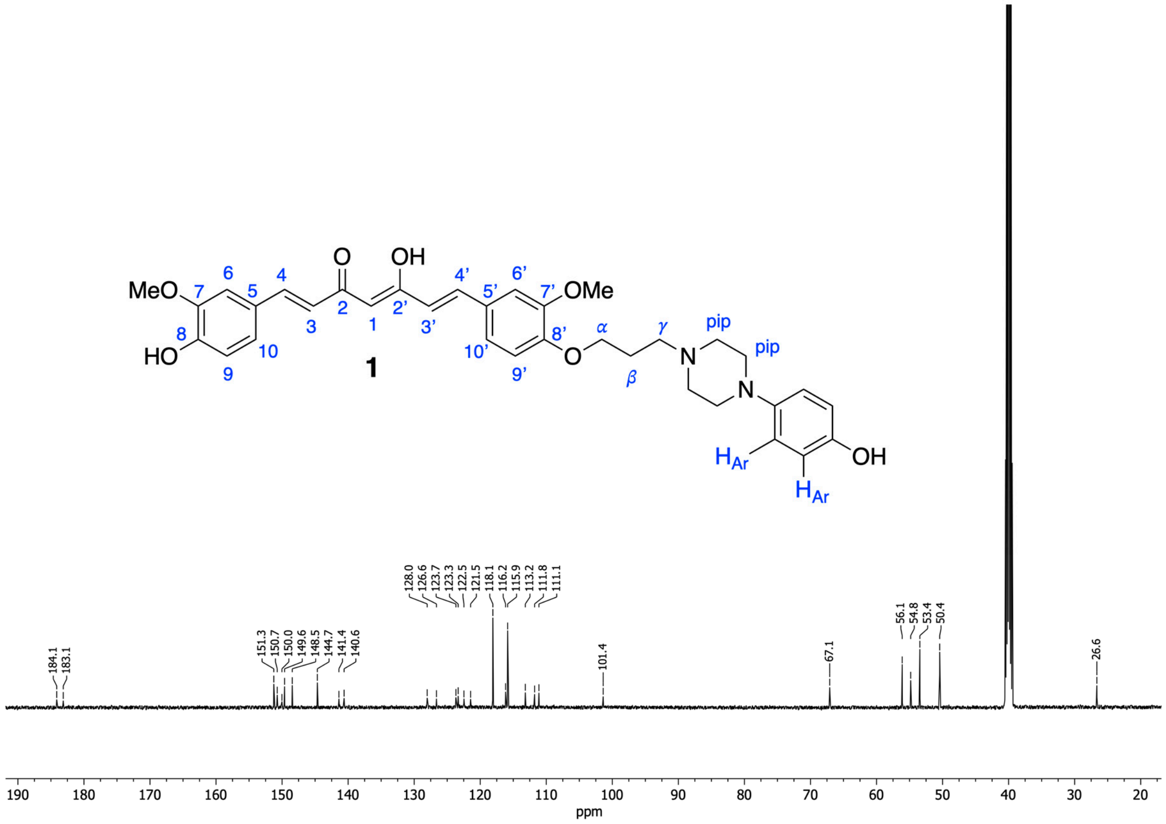

Figure S12. ^{13}C-NMR of compound **1** in DMSO-*d6*

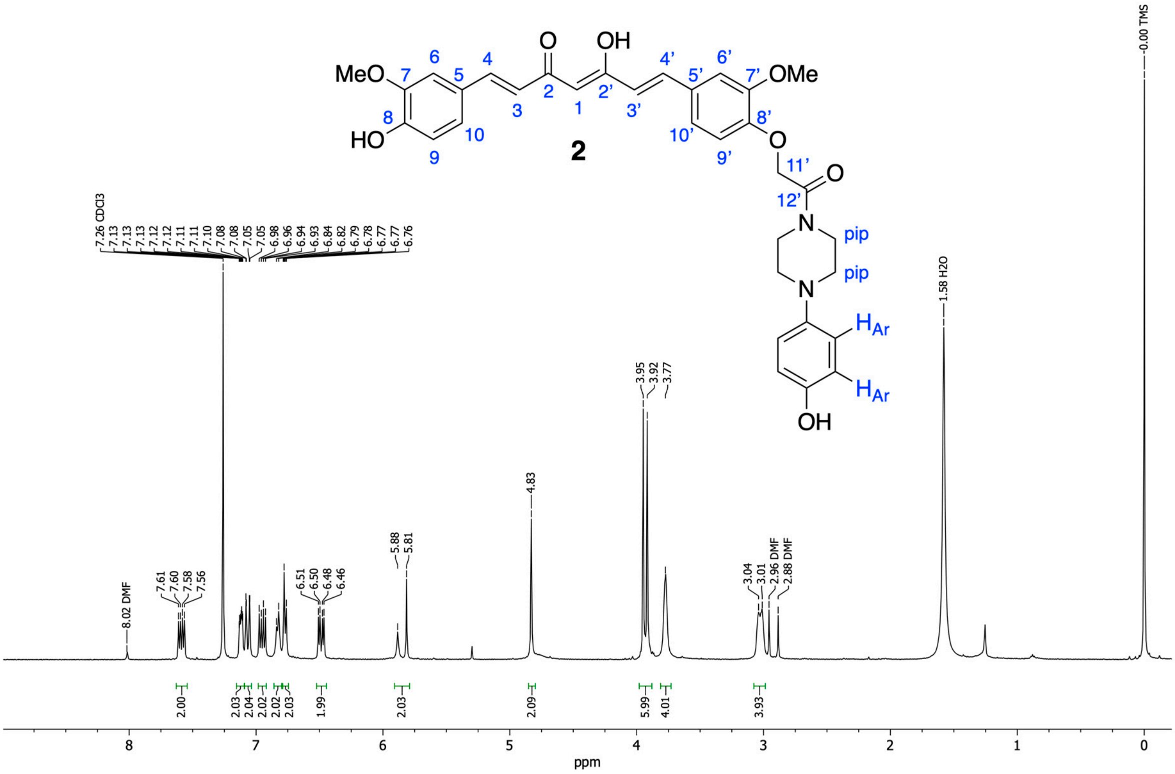

Figure S13. ^{1}H-NMR of compound **2** in $CDCl_3$

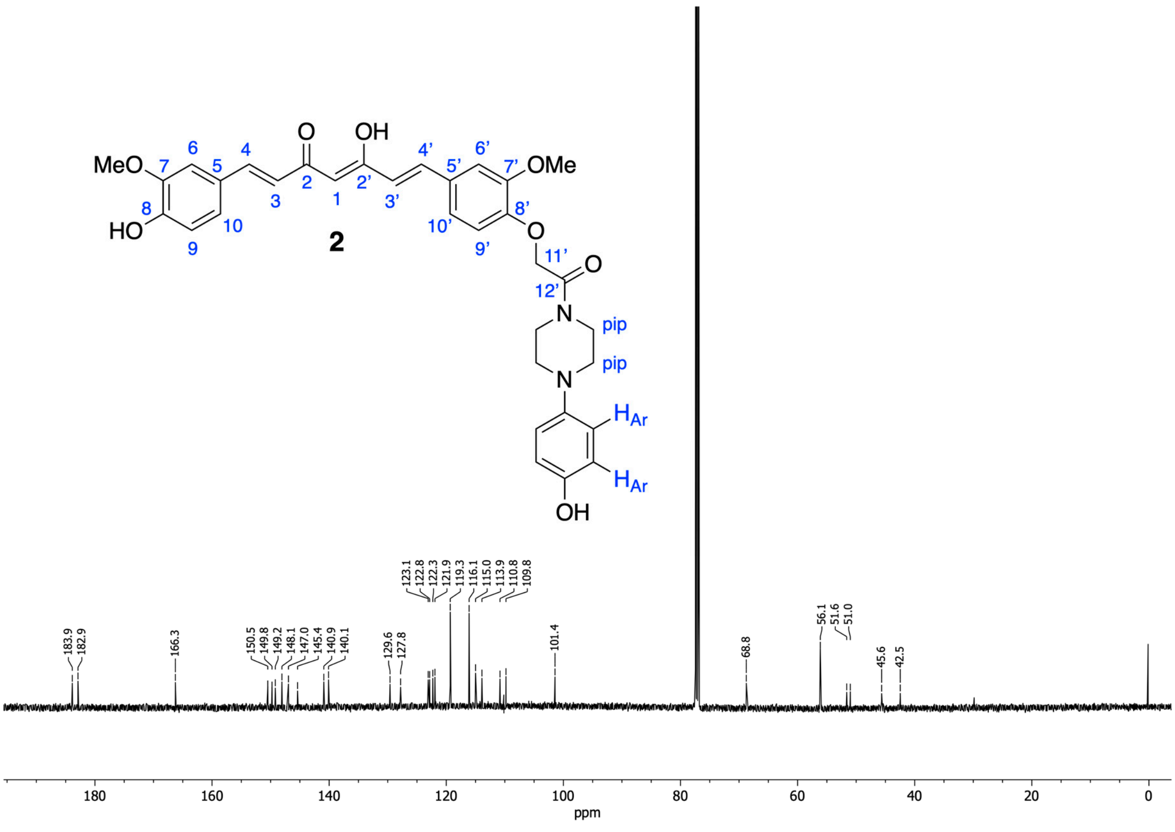

Figure S14. ^{13}C-NMR of compound **2** in $CDCl_3$

Photophysical characterization of CUR-C3-PP 1 and CUR-CO-PP 2 *vs* CUR

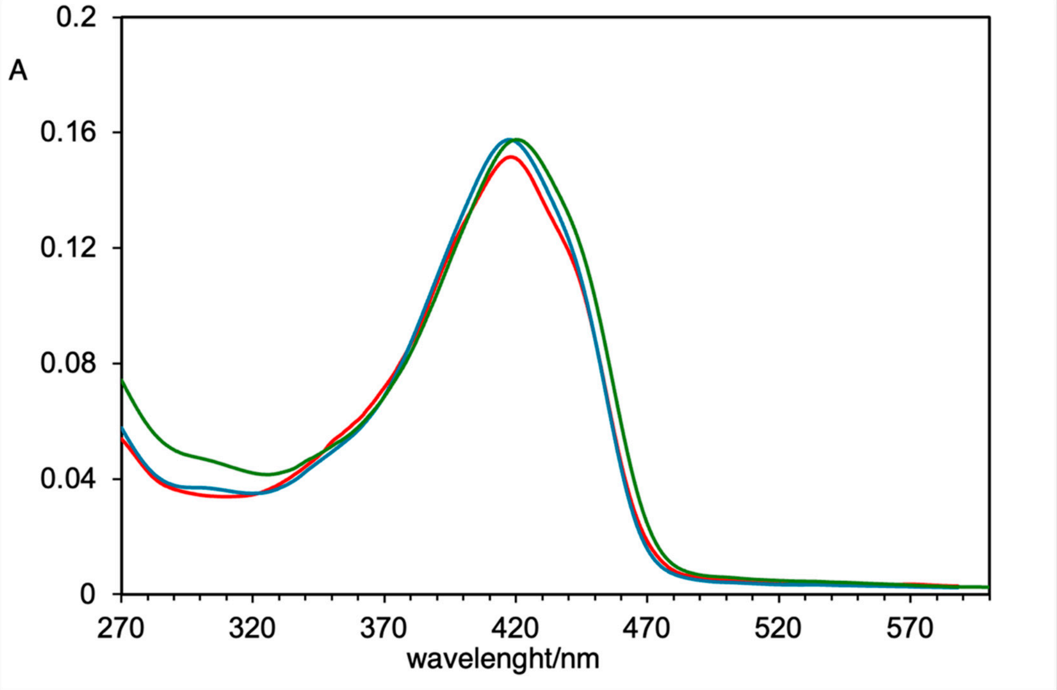

Figure S15. Absorption spectra of CUR (red), **Cur-C3-PP 1** (green) and **CUR-CO-PP 2** (blue) recorded in DCM at a concentration of 4.3 x 10^{-6} M.

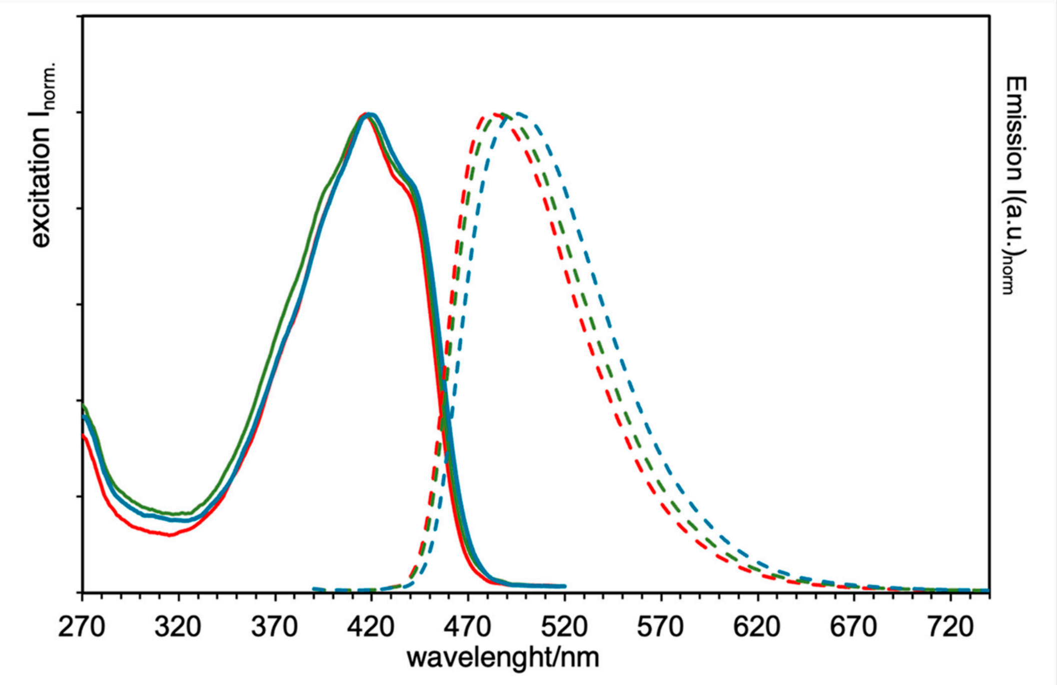

Figure S16. Normalized excitation (solid lines, λem = 520 nm) and emission (dashed lines) spectra of CUR (red), **Cur-C3-PP 1** (green) and **CUR-CO-PP 2** (blue) recorded in DCM.

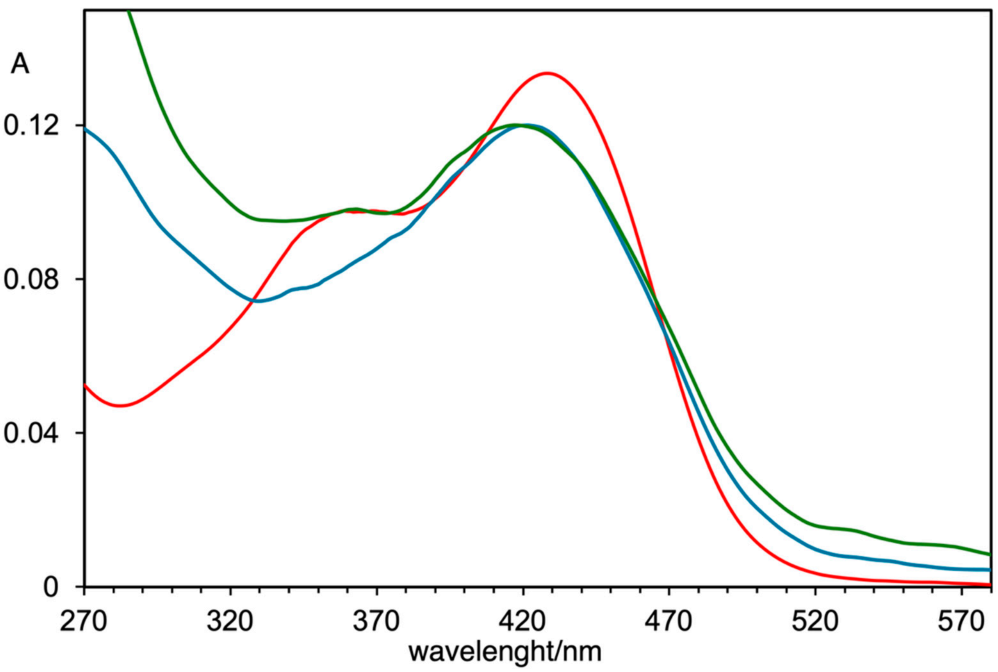

Figure S17. Absorption spectra of CUR (red), **Cur-C3-PP 1** (green) and **CUR-CO-PP 2** (blue) recorded in phosphate-buffered saline (PBS) containing 1% DMSO at a concentration of 4.3 x 10^{-6} M.

Fluorescence imaging studies and cytotoxicity studies

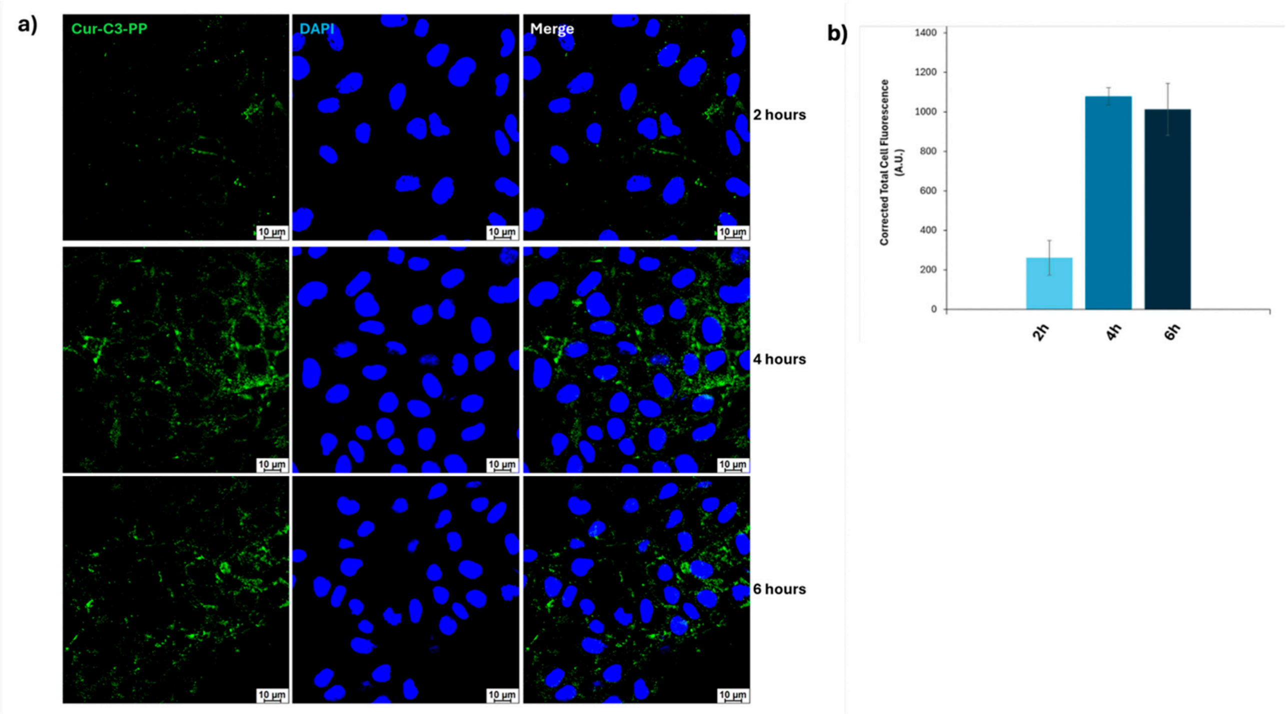

Figure S18. a) Representative confocal microscopy images of B16-F10 cells after 2h, 4h, and 6h of incubation with 10 uM of **CUR-C3-PP 1**. Scale bar = 10 µm. b) Quantification of cellular uptake expressed as Corrected Total Cell Fluorescence (CTCF). Data represent mean ± SEM (n=3).

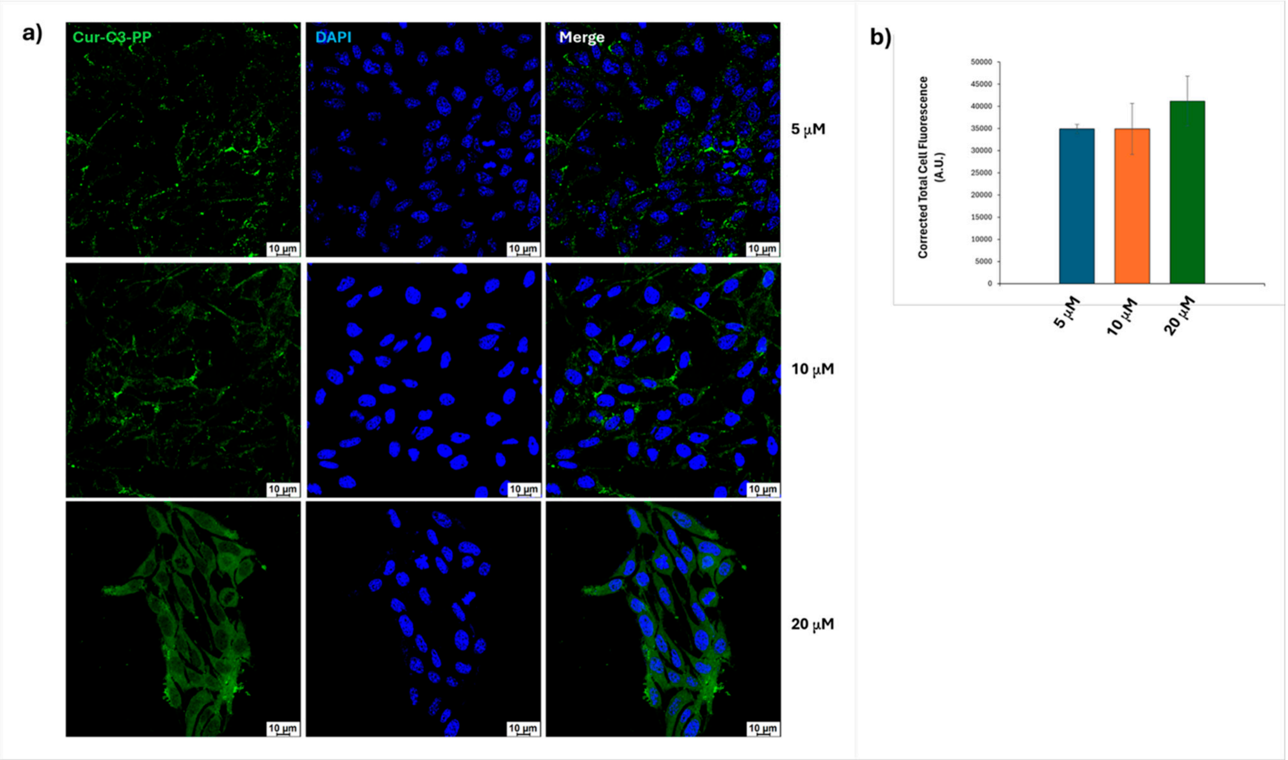

Figure S19. a) Representative confocal microscopy images of B16-F10 cells treated with 5, 10, and 20 µM of **Cur-C3-PP 1** for 24 h. Scale bar = 10 µm. b) Quantification of cellular uptake expressed as Corrected Total Cell Fluorescence (CTCF). Data represent mean ± SEM (n=3).

References

18. Spath, A.; Platzer, K.; Maisch, T.; Eichner, A. 1,7-Diaryl-1,6-heptadiene-3,5-dione derivatives, method for manufacture, and use thereof. US11191855B2, 7 December 2021.

19. Ryu, E.K.; Choe, Y.S.; Lee, K.H.; Choi, Y.; Kim, B.T. Curcumin and dehydrozingerone derivatives: synthesis, radiolabeling, and evaluation for beta-amyloid plaque imaging. *J Med Chem* **2006**, *49*, 6111-6119, doi:10.1021/jm0607193.

21. Mirabile, S.; Ielo, L.; Lombardo, L.; Ricci, F.; Gitto, R.; Germanò, M.P.; Pace, V.; De Luca, L. Leveraging the 3-Chloro-4-fluorophenyl Motif to Identify Inhibitors of Tyrosinase from Agaricus bisporus. *Int J Mol Sci* **2023**, *24*, 7944, doi:10.3390/ijms24097944.

27. Ismaya, W.T.; Rozeboom, H.J.; Weijn, A.; Mes, J.J.; Fusetti, F.; Wichers, H.J.; Dijkstra, B.W. Crystal structure of Agaricus bisporus mushroom tyrosinase: identity of the tetramer subunits and interaction with tropolone. *Biochemistry* **2011**, *50*, 5477-5486, doi:10.1021/bi200395t.
